# DLL4^+^ neutrophils promote Notch1-mediated endothelial PANoptosis to exacerbate acute lung injury in sepsis

**DOI:** 10.1172/JCI194310

**Published:** 2025-12-15

**Authors:** Hui Jin, Saoirse Holland, Alok Jha, Gaifeng Ma, Jingsong Li, Atsushi Murao, Monowar Aziz, Ping Wang

**Affiliations:** 1 Center for Immunology and Inflammation, Feinstein Institutes for Medical Research, Manhasset, New York, USA.; 2 Departments of Surgery and Molecular Medicine, Zucker School of Medicine, Manhasset, New York, USA.

**Keywords:** Immunology, Inflammation, Neutrophils

## Abstract

Neutrophils play a critical role in sepsis-induced acute lung injury (ALI). Extracellular cold-inducible RNA-binding protein (eCIRP), a damage-associated molecular pattern, promotes neutrophil heterogeneity. While delta-like ligand 4 (DLL4) expression has been studied in various cell populations, its expression in neutrophils and impact on inflammation remain unknown. Here, we discovered that eCIRP induces DLL4^+^ neutrophils. These neutrophils trigger PANoptosis, a novel proinflammatory form of cell death initiated by Z-DNA–binding protein-1 (ZBP1) in pulmonary vascular endothelial cells (PVECs). In sepsis, DLL4^+^ neutrophils increase in the blood and lungs, upregulating ZBP1, cleaved gasdermin D, cleaved caspase-3, and phosphorylated MLKL, all of which are markers of PANoptosis, exacerbating ALI. DLL4 binds to Notch1 on PVECs and activates Notch1 intracellular domain to increase ZBP1-mediated endothelial PANoptosis. We discovered what we believe to be a novel Notch1-DLL4 inhibitor (NDI), derived from Notch1 to specifically block this interaction. Our findings reveal that NDI reduced endothelial PANoptosis in vitro and in vivo, attenuated pulmonary injury induced by DLL4^+^ neutrophils, and decreased lung water content and permeability, indicating improved barrier function. NDI also reduced serum injury and inflammatory markers and improved survival rate in sepsis. These findings underscore the Notch1-DLL4 pathway’s critical role in DLL4^+^ neutrophil–mediated ALI. Targeting the Notch1-DLL4 interaction with an NDI represents a promising therapeutic strategy for sepsis-induced ALI.

## Abstract 2

In this study, we report the discovery of a unique neutrophil subpopulation with high DLL4 expression, induced by extracellular cold-inducible RNA-binding protein (eCIRP)—a key DAMP in sepsis. These DLL4⁺ neutrophils significantly induce pulmonary endothelial cell PANoptosis, exacerbate acute lung injury, and amplify systemic inflammation in sepsis.

## Graphical abstract

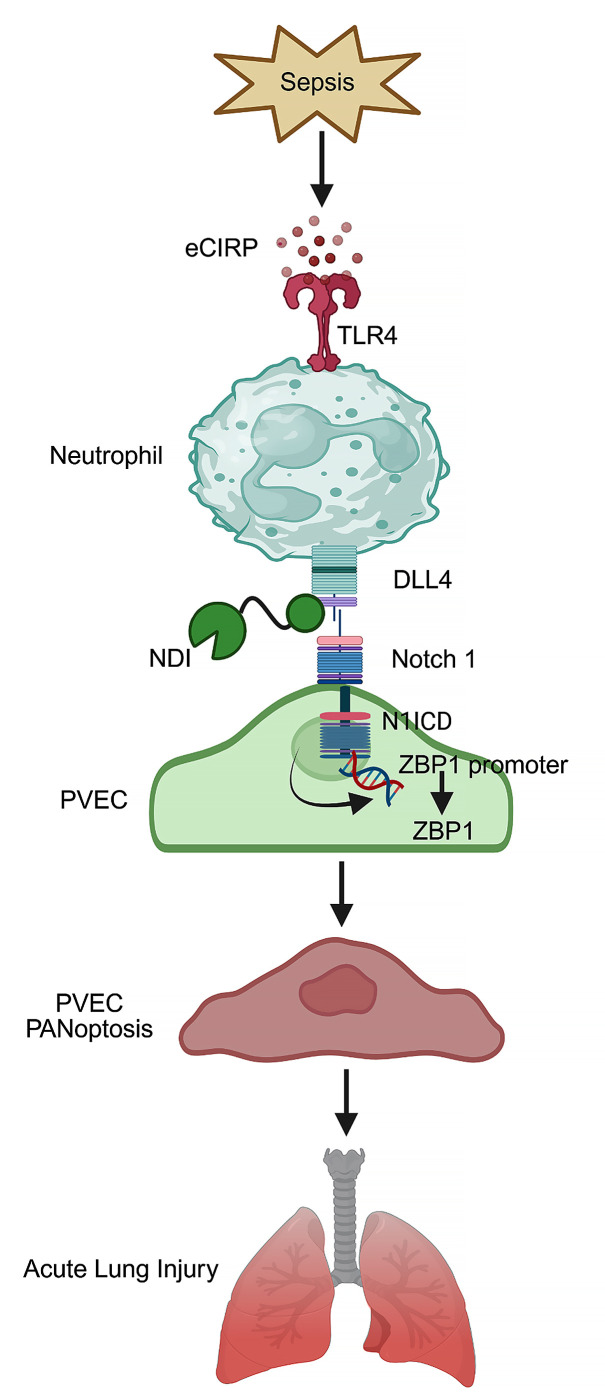

## Introduction

Sepsis is caused by an exaggerated immune response to an infection (1). Neutrophils are the primary responders to infection (2). However, uncontrolled activation of neutrophils exacerbates inflammation, endothelial dysfunction, and tissue injury, contributing to increased mortality in sepsis (3). Neutrophils exist in various subtypes or functional states, which differ in critical immune phenotypes, including their release of cytokines, myeloperoxidase, reactive oxygen species (ROS), and neutrophil extracellular traps (NETs) under different pathological conditions (3). Acute lung injury (ALI) and its severe manifestation acute respiratory distress syndrome are triggered by various intrapulmonary and extrapulmonary factors (4), including pulmonary contusion, sepsis, trauma, burns, blood transfusion, and infection (5). The activation and recruitment of neutrophils produce proinflammatory cytokines, ROS, granular enzymes, and NETs, which are important to the progression of ALI (6). In addition to these effector molecules, neutrophils undergo various phenotypic changes in response to various stimuli (7). These changes enable neutrophils to exert novel, complex functions that influence the lung microenvironment and contribute to ALI in sepsis (8).

Damage-associated molecular patterns (DAMPs) are key drivers of neutrophil activation and heterogeneity, potentially worsening outcomes in sepsis (6, 9). Notably, our previous studies have revealed extracellular cold-inducible RNA-binding protein (eCIRP) as a critical DAMP, exacerbating organ injury and mortality in sepsis (10). Elevated serum levels of eCIRP have been correlated with sepsis severity and mortality in patients (11, 12). While traditionally known as an RNA chaperone residing within the nuclear compartment, CIRP undergoes cytoplasmic translocation and subsequent extracellular release in response to stimuli such as lipopolysaccharides or hypoxia (13). Once released, eCIRP augments inflammation by binding to Toll-like receptor 4 (TLR4) and triggering receptor expressed on myeloid cells-1 (TREM-1) on macrophages and neutrophils (14–16).

PANoptosis integrates three inflammatory cell death pathways — pyroptosis, apoptosis, and necroptosis — into a single process (17). Z-DNA–binding protein-1 (ZBP1), an innate immune sensor of nucleic acids, triggers PANoptosis by activating RIP3, caspase-8, and NLRP3 (18). This integration of multiple cell death pathways enables PANoptosis to elicit a robust immune response, effectively eliminating infected or damaged cells (19). However, PANoptosis by itself can lead to excessive inflammation, contributing to the development of various infectious and inflammatory diseases, highlighting its significance and distinct role in disease pathophysiology (20, 21). Moreover, research indicates that PANoptosis can be triggered not only by Z-DNA but also by the cytokine storm that occurs during sepsis. For instance, during different infectious diseases, TNF-α and IFN-γ work together to sensitize inflammatory cells to undergo PANoptosis, which plays a critical role in the development of ALI (22). However, the role of PANoptosis in pulmonary endothelial cell injury, and its induction by neutrophils in sepsis, along with its underlying mechanisms, remain underexplored.

The Notch system is composed of four Notch receptors (Notch1–4) and four Notch ligands, Delta-like ligand 1 (DLL1), DLL4, Jagged1 (Jag1), and Jag2, in mammals (23). The interactions between receptors and ligands trigger proteolytic processing and the nuclear translocation of the Notch1 intracellular domain (N1ICD), which then associates with a CBF1/Su(H)/Lag-1 (CSL) family transcription factor to drive target gene expression (24). The Notch signaling pathway is recognized for its broad regulatory roles in cell fate determination, and it has emerged as a pivotal player in inflammatory cell death and inflammation. Notably, endothelial cells predominantly express Notch1 and Notch4, which are integral in regulating angiogenesis and arterial specification within the vascular system (25). Notch1-DLL4 signaling is critical for facilitating cell-cell communication, activating NF-κB, and driving inflammatory responses (25, 26).

In this study, we investigated how eCIRP induces DLL4 expression on neutrophils to promote endothelial cell PANoptosis in sepsis. We also explored the mechanisms by which DLL4^+^ neutrophils exacerbate endothelial barrier dysfunction and ALI and demonstrated the potential of developing an inhibitor to mitigate DLL4^+^ neutrophil–induced endothelial cell PANoptosis, aiming to reduce sepsis severity.

## Results

### eCIRP stimulates neutrophil DLL4 expression via TLR4, leading to the elevated DLL4^+^ neutrophil phenotype in the blood and lungs in sepsis.

**Figure 1:**
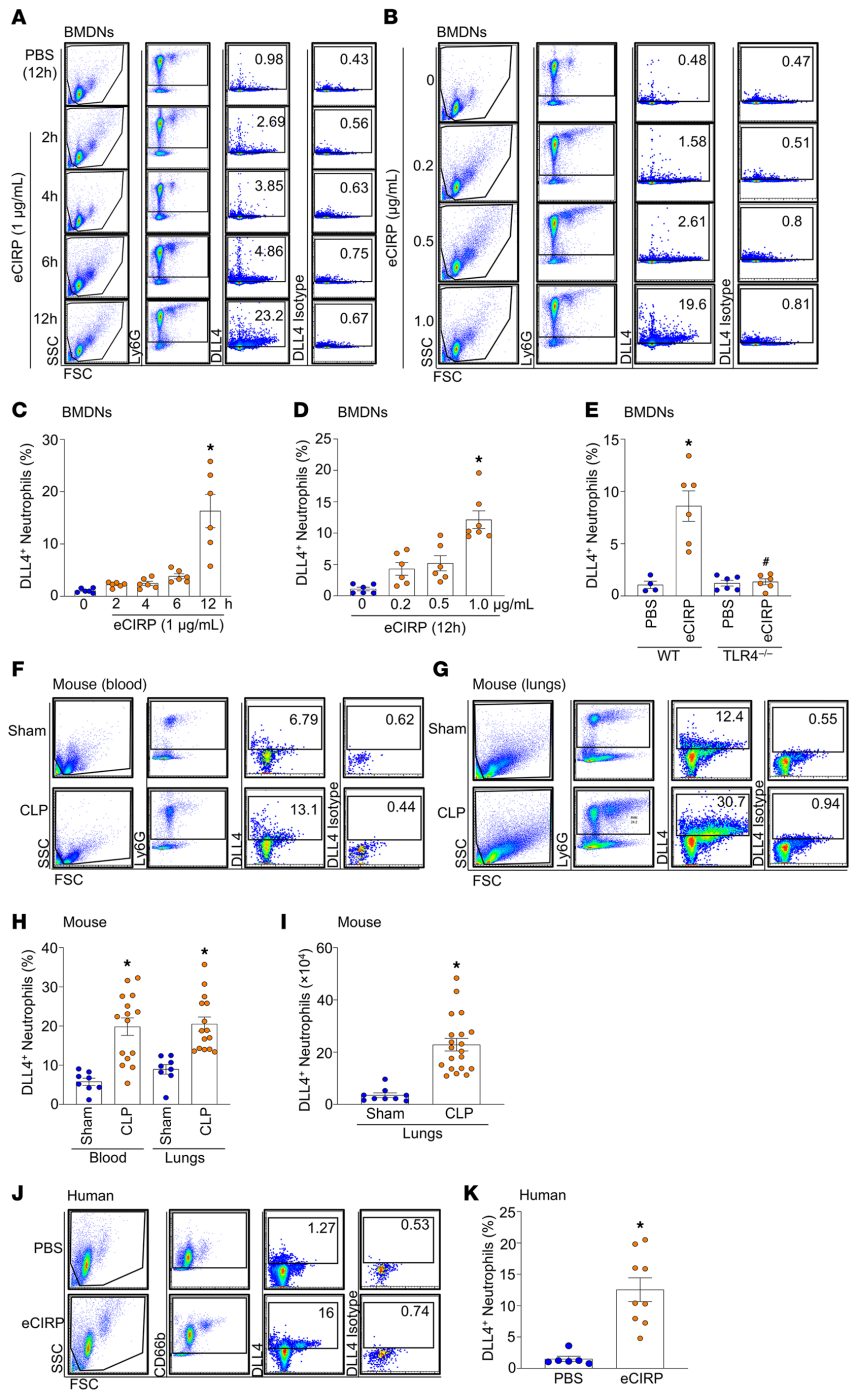
Treatment with eCIRP generates DLL4^+^ neutrophils in vitro and in vivo. (**A** and **B**) Bone marrow–derived neutrophils (BMDNs) (1 × 10^6^/mL) collected from wild-type (WT) mice were treated with eCIRP for various times (**A**) and at various doses (**B**). The frequency of DLL4^+^ neutrophils (Ly6G^+^DLL4^+^) was then assessed by flow cytometry. (**C** and **D**) Bar graph of percentage of DLL4^+^ neutrophils. Experiments were performed at least 3 times, and all data were analyzed. Data are expressed as means ± SEM and were analyzed using 1-way ANOVA. *n* = 4–7 per group. *P < 0.05 vs. PBS. (**E**) Mouse BMDNs (1 × 10^6^/mL) collected from WT and TLR4^–/–^ were treated with 1 μg/mL eCIRP for 12 hours; the bar graph shows the frequency of DLL4^+^ neutrophils (Ly6G^+^DLL4^+^), assessed by flow cytometry. Data are expressed as means ± SEM and were analyzed using 1-way ANOVA. *n* = 4–6 per group. *P < 0.05 vs. WT-PBS. ^#^*P* < 0.05 vs. WT-eCIRP. Sepsis was induced in WT mice by cecal ligation and puncture (CLP). (**F** and **G**) After 20 hours, the frequency of DLL4^+^ neutrophils (Ly6G^+^DLL4^+^) in the blood and lungs was assessed by flow cytometry. (**H** and **I**) Bar graphs of percentage and cell number of DLL4^+^ neutrophils. Experiments were performed at least 3 times, and all data were analyzed. Data are expressed as means ± SEM and were analyzed using 1-way ANOVA. *n* = 8–20 mice per group. *P < 0.05 vs. sham. (**J** and **K**) Human peripheral blood neutrophils (1 × 10^6^/mL) obtained from healthy volunteers were treated with eCIRP (1 μg/mL) for 12 hours. The frequency of DLL4^+^ neutrophils (CD66^+^DLL4^+^) was then assessed by flow cytometry. Data reflecting at least 3 independent experiments are expressed as means ± SEM and were analyzed using 1-way ANOVA. *n* = 6–9 per group. *P < 0.05 vs. PBS.

We found that under normal conditions, less than 1% of neutrophils expressed DLL4 on their surface, while eCIRP treatment significantly increased the frequency (percentage) of DLL4^+^ neutrophils in a time- and dose-dependent manner (Figure 1, A–D). Treatment with 1 μg/mL eCIRP for 12 hours resulted in a greater than 10-fold increase in DLL4^+^ neutrophils compared with baseline. Interestingly, eCIRP-mediated induction of DLL4^+^ neutrophils was significantly abrogated in TLR4^–/–^ neutrophils (Figure 1E), indicating that eCIRP induces DLL4^+^ neutrophils via TLR4. To assess the status of DLL4^+^ neutrophils in sepsis, we performed a cecal ligation and puncture (CLP) model of sepsis. At 20 hours after CLP, there was a significant increase in both the percentage and the number of DLL4^+^ neutrophils in the blood and lungs of septic mice compared with sham (Figure 1, F–I). Thus DLL4^+^ neutrophils were markedly elevated in sepsis and may contribute to ALI pathophysiology. In line with the mouse data, eCIRP also significantly increased the frequency of DLL4^+^ neutrophils in human peripheral blood neutrophils (Figure 1, J and K). These data suggest that eCIRP stimulation of neutrophils generates a novel DLL4^+^ neutrophil population in both mice and humans.

### DLL4^+^ neutrophils induce pulmonary endothelial cell PANoptosis in sepsis.

**Figure 2:**
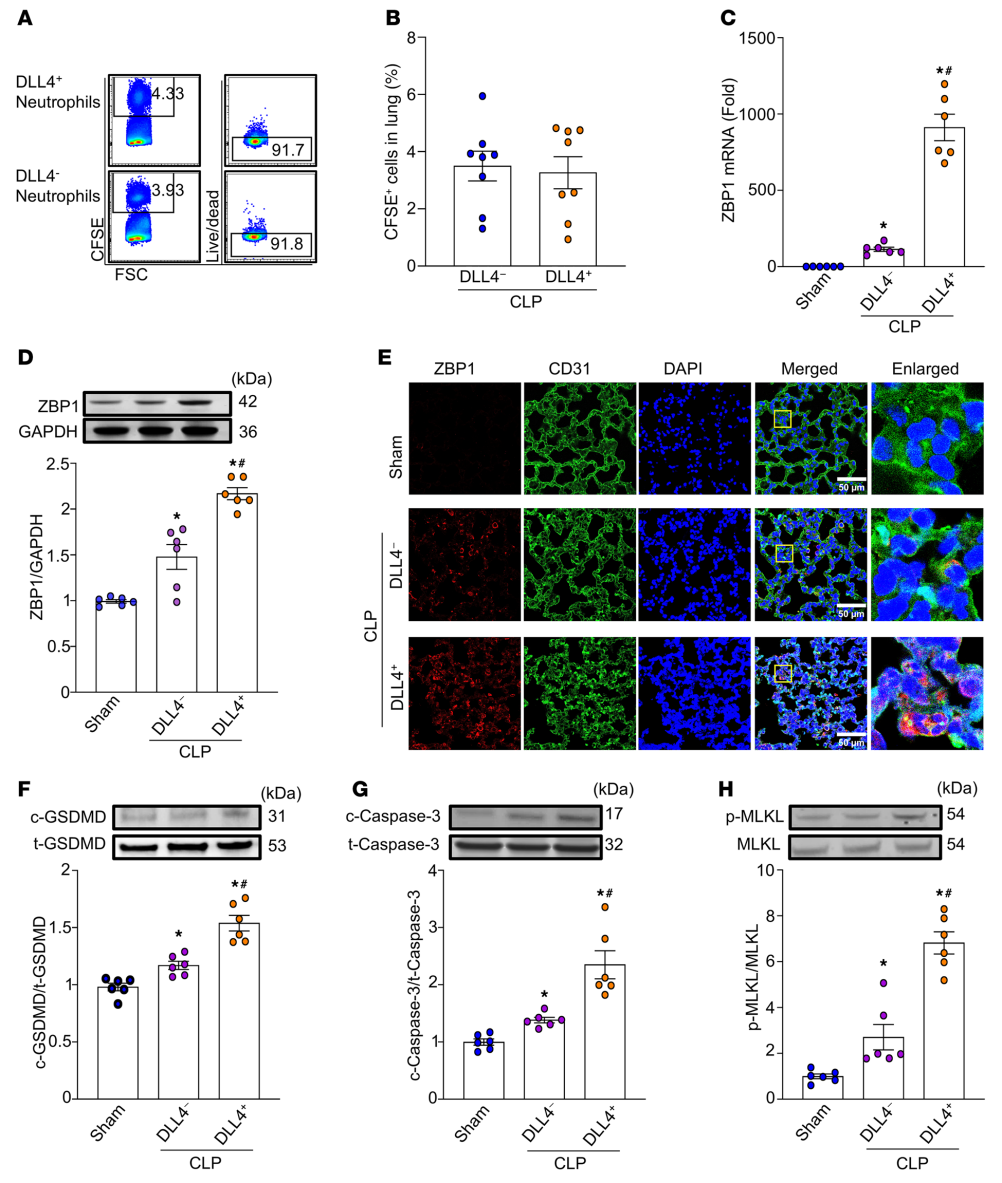
DLL4^+^ neutrophils induce lung endothelial cell PANoptosis in sepsis. BMDNs isolated from wild-type mice were stimulated with eCIRP for 2 hours. FACS-sorted DLL4^+^ neutrophils and DLL4^–^ neutrophils (1 × 10^6^/100 mL/mouse) with or without CFSE label were then delivered into mice via retro-orbital injection at the time of CLP. At 20 hours, the lungs were harvested. (**A** and **B**) CFSE^+^ neutrophils and their viability were analyzed by flow cytometry. Experiments were performed at least 3 times, and all data were analyzed. Data are expressed as means ± SEM and were analyzed using 1-way ANOVA. *n* = 8 per group. (**C**) Total RNA was extracted from lungs, and ZBP1 expression was analyzed by RT-PCR. Experiments were performed at least 3 times, and all data were analyzed. Data are expressed as means ± SEM and were analyzed using 1-way ANOVA. *n* = 6 per group. (**D** and **F**–**H**) Western blot analysis was performed to measure protein levels of ZBP1 in total lung tissues and cleaved gasdermin D (c-GSDMD), total GSDMD (t-GSDMD), cleaved caspase-3 (c-caspase-3), total caspase-3 (t-caspase-3), phosphorylated MLKL (p-MLKL), and MLKL in lung. Pyroptosis, apoptosis, and necroptosis (PANoptosis) were quantified as c/t GSDMD, c/t caspase-3, and p-MLKL/MLKL. Experiments were performed at least 3 times, and all data were analyzed. Data are expressed as means ± SEM and were analyzed using 1-way ANOVA. *n* = 6 per group. (**E**) Confocal microscopy was used to assess ZBP1 expression in lungs. ZBP1 was stained red, pulmonary endothelial cells were stained green (CD31), and nuclei were stained blue (DAPI). Scale bars: 50 μm. *P < 0.05 vs. sham. ^#^*P* < 0.05 vs. DLL4^–^ neutrophils.

To investigate the role of DLL4^+^ neutrophils in sepsis-induced ALI, we assessed their impact on lung endothelial cells. Specifically, we hypothesized that DLL4^+^ neutrophils induce endothelial PANoptosis, a form of programmed cell death regulated by ZBP1. To test this, we adoptively transferred DLL4^+^ or DLL4^–^ neutrophils into the mice concurrent with CLP-induced sepsis. After 20 hours post-CLP, both DLL4^+^ and DLL4^–^ neutrophils were detected in the lungs and exhibited over 90% viability following adoptive transfer (Figure 2, A and B). We found that septic mice receiving DLL4^+^ neutrophils showed significantly higher ZBP1 mRNA and protein expression compared with sham-operated mice or mice receiving DLL4^–^ neutrophils (Figure 2, C and D). We further isolated CD31^+^ pulmonary endothelial cells and analyzed ZBP1 expression within this population. The results showed that ZBP1 was markedly upregulated in pulmonary endothelial cells from CLP mice treated with DLL4^+^ neutrophils, compared with control groups (Supplemental Figure 1; supplemental material available online with this article; https://doi.org/10.1172/JCI194310DS1). Confocal microscopy of lung tissues revealed the elevated ZBP1 protein expression in pulmonary endothelial cells in the DLL4^+^ neutrophil–injected septic mice (Figure 2E). Given that PANoptosis involves pyroptosis, apoptosis, and necroptosis, we assessed markers of each pathway: pyroptosis by detecting cleaved gasdermin D (c-GSDMD), apoptosis by detecting cleaved caspase-3 (c-caspase-3), and necroptosis by detecting phosphorylated mixed-lineage kinase domain–like pseudokinase (p-MLKL). Mice injected with DLL4^+^ neutrophils exhibited significantly increased levels of c-GSDMD, c-caspase-3, and p-MLKL compared with both sham and DLL4^–^ neutrophil–injected septic mice (Figure 2, F–H). These findings suggest that DLL4^+^ neutrophils enhanced PANoptosis in the pulmonary endothelium in sepsis.

### DLL4^+^ neutrophils promote endothelial PANoptosis via Notch1-DLL4 axis.

**Figure 3:**
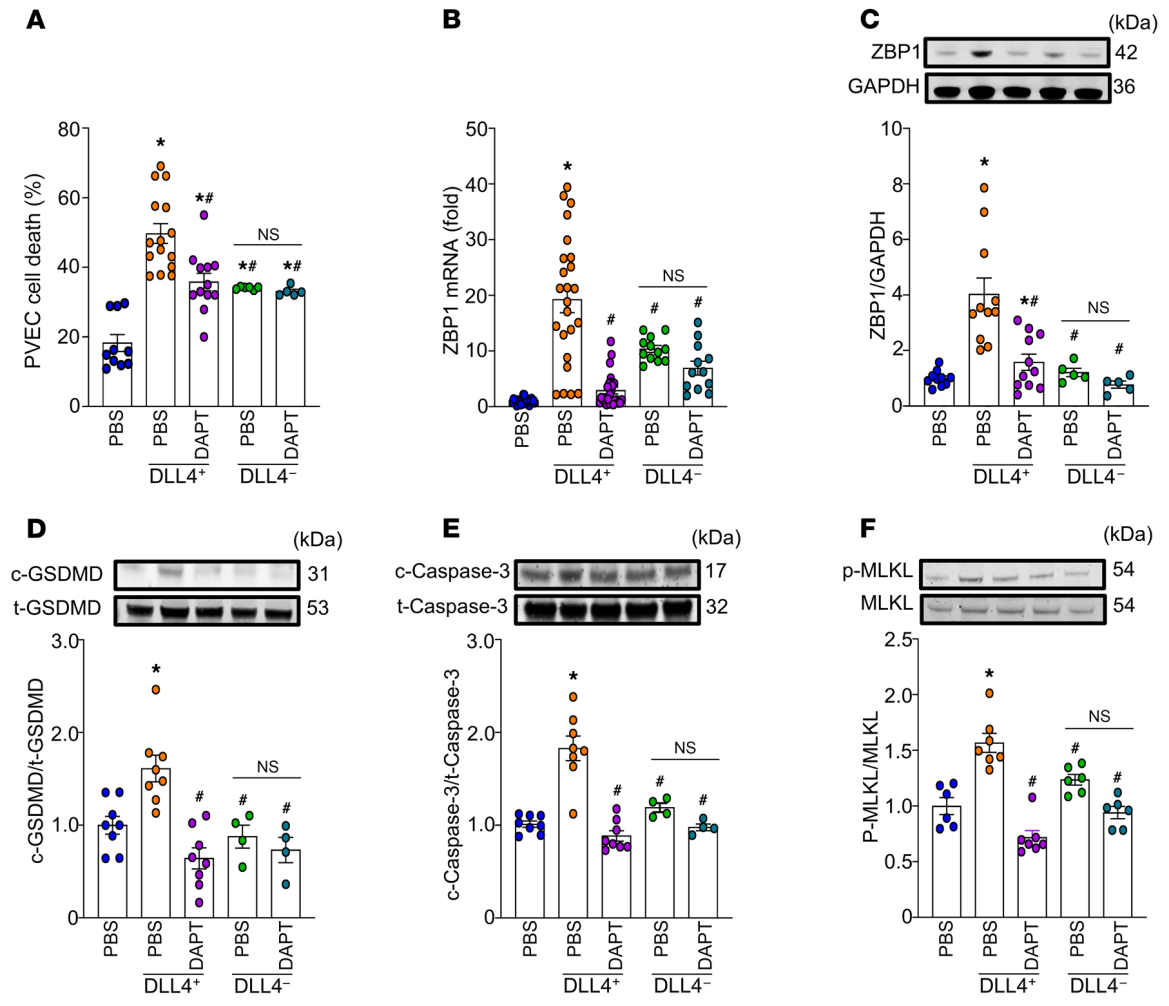
DAPT attenuates endothelial PANoptosis by inhibiting Notch1-DLL4 interaction. PVECs (0.5 × 10^6^) were cocultured with sorted DLL4^+^ (0.5 × 10^6^) neutrophils or DLL4^–^ (0.5 × 10^6^) neutrophils, then treated with DAPT or PBS. After 16 hours, cell viability was assessed using propidium iodide staining. (**A**) Cell death was quantified as the percentage of propidium iodide–positive cells. *n* = 6–15 per group. Data are representative of 3 independent experiments. Data are expressed as means ± SEM and were analyzed using 1-way ANOVA. (**B**) RNA was extracted, and ZBP1 expression was analyzed by RT-PCR. Experiments were performed at least 3 times, and all data were analyzed. Data are expressed as means ± SEM and were analyzed using 1-way ANOVA. *n* = 11–25 per group. *P < 0.05 vs. PBS. ^#^*P* < 0.05 vs. DLL4^+^ neutrophils/PBS. (**C**–**F**) Western blot analysis was performed to measure protein levels of ZBP1, c-GSDMD, t-GSDMD, c-caspase-3, t-caspase-3, p-MLKL, and MLKL in PVECs. Pyroptosis, apoptosis, and necroptosis were quantified as c/t GSDMD, c/t caspase-3, and p-MLKL/MLKL. *n* = 4–12 per group. Data are representative of 3 independent experiments. Data are expressed as means ± SEM and were analyzed using 1-way ANOVA. *P < 0.05 vs. PBS. ^#^*P* < 0.05 vs. DLL4^+^ neutrophils.

We then aimed to elucidate the mechanism by which DLL4^+^ neutrophils induce endothelial cell PANoptosis. DLL4^+^ and DLL4^–^ neutrophils sorted by flow cytometry were cocultured with pulmonary vascular endothelial cells (PVECs) to examine endothelial cell viability. Our results showed a significant increase in propidium iodide–positive PVECs when cocultured with DLL4^+^ neutrophils compared with control PVECs cultured without DLL4^+^ neutrophils and PVECs cultured with DLL4^–^ neutrophils, indicating that DLL4^+^ neutrophils promote PVEC death (Figure 3A). We found that DLL4^+^ neutrophils directly activated Notch1 signaling in PVECs, resulting in increased expression of the Notch1 intracellular domain (N1ICD) (Supplemental Figure 2). *N*-[*N*-(3,5-difluorophenacetyl)-l-alanyl]-*S*-phenylglycine t-butyl ester (DAPT), a Notch1 chemical inhibitor, specifically inhibits Notch1 signaling by acting as a γ-secretase inhibitor, preventing the cleavage of the Notch receptor, which is necessary for the release of N1ICD (23). Our results showed that DAPT treatment resulted in 33% reduction in PVEC death compared with the untreated DLL4^+^ neutrophil cocultures (Figure 3A), suggesting that Notch1-DLL4 signaling is a critical pathway of DLL4^+^ neutrophil–induced PVEC death. We also found that ZBP1 mRNA expression was significantly increased in the DLL4^+^ neutrophil–PVEC cocultures, while DAPT reduced it to the baseline levels (Figure 3B). Additionally, DAPT treatment significantly reduced the elevated expression of PANoptosis markers, ZBP1, c-GSDMD, c-caspase-3, and p-MLKL, in DLL4^+^ neutrophil–stimulated PVECs (Figure 3, C–F). However, DAPT treatment did not significantly reduce ZBP1, c-GSDMD, c-caspase-3, or p-MLKL levels in DLL4^–^ neutrophil–treated PVECs. These results revealed that DLL4^+^ neutrophils promote PVEC PANoptosis through the Notch1-DLL4 pathway.

### Notch1-DLL4 inhibitor decreases DLL4^+^ neutrophil–induced endothelial PANoptosis.

**Figure 4:**
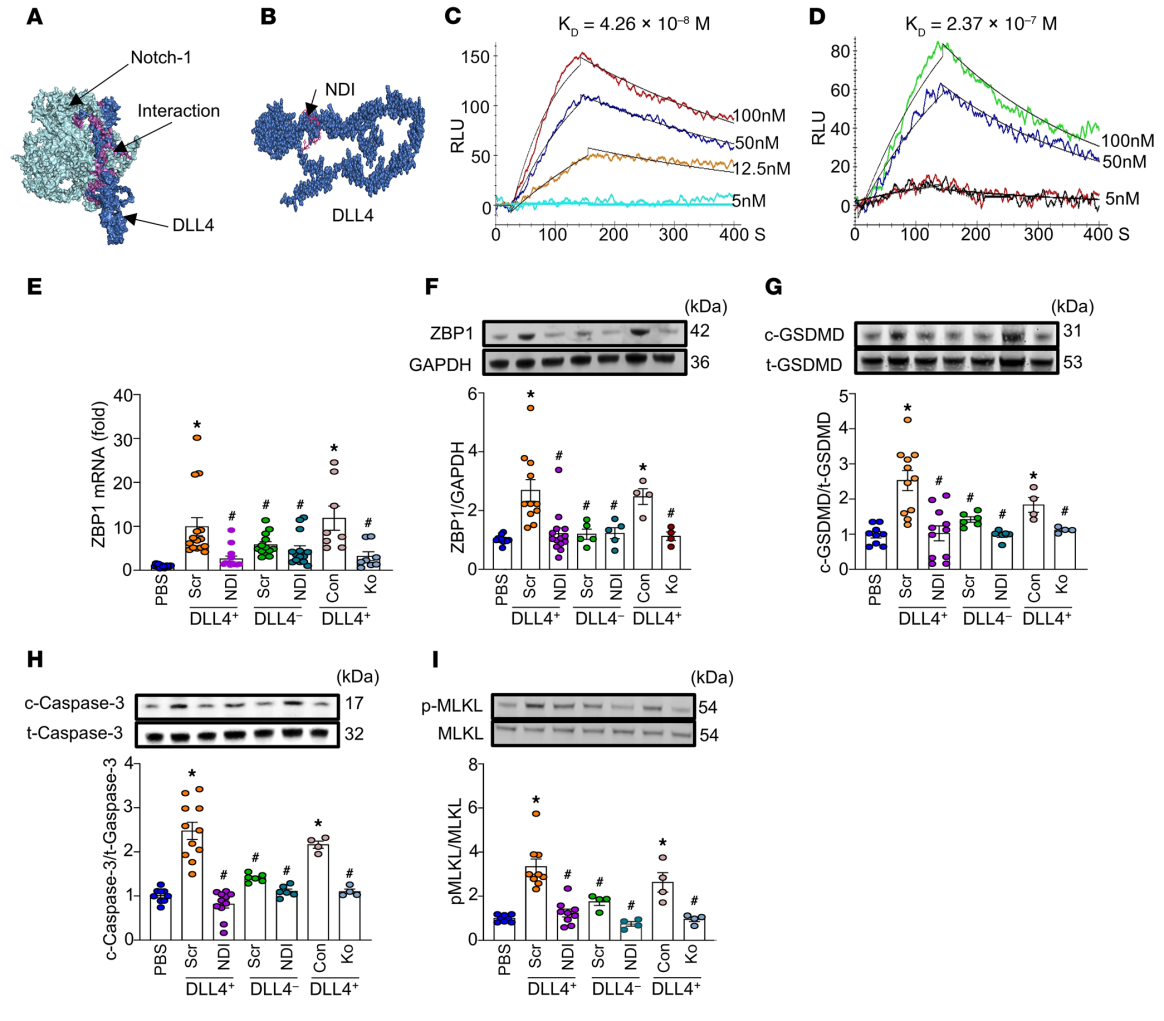
Development and evaluation of small peptide NDI attenuates PVEC PANoptosis by inhibiting Notch1-DLL4 interaction. (**A**) Computational modeling for the protein-protein interaction analysis between Notch1 extracellular domain and DLL4. Notch1 is shown in green, DLL4 in blue, and the interaction between Notch1 and DLL4 in purple. (**B**) Computational modeling was used to design Notch1-DLL4 inhibitor (NDI) derived from the Notch1 sequence that binds to DLL4. DLL4 is shown in blue, NDI in red. (**C** and **D**) BIAcore analysis was conducted to determine the binding affinity (*K_D_* value) of DLL4 to Notch1 and the inhibitory effect of NDI on this interaction. Wild-type PVECs (0.5 × 10^6^) were cocultured with sorted DLL4^+^ neutrophils (0.5 × 10^6^) or DLL4^–^ neutrophils (0.5 × 10^6^), then treated with either 10 μM scramble or NDI. PVECs (0.5 × 10^6^) were first treated with Notch1-knockdown plasmid or its negative control for 24 hours. These PVECs were then cocultured with sorted DLL4^+^ neutrophils (0.5 × 10^6^). (**E**) After 16 hours, total RNA was extracted and ZBP1 expression was analyzed by RT-PCR. Experiments were performed at least 3 times, and all data were analyzed. Data are expressed as means ± SEM and were analyzed using 1-way ANOVA. *n* = 8–15 per group. (**F**–**I**) Western blot analysis was performed to measure protein levels of ZBP1, c-GSDMD, t-GSDMD, c-caspase-3, t-caspase-3, p-MLKL, and MLKL in PVECs. Pyroptosis, apoptosis, and necroptosis were quantified as c/t GSDMD, c/t caspase-3, and p-MLKL/MLKL. *n* = 6–14 per group. Data are representative of 3 independent experiments. Data are expressed as means ± SEM and were analyzed using 1-way ANOVA. *P < 0.05 vs. PBS. ^#^*P* < 0.05 vs. DLL4^+^ neutrophils/scramble. Con, Notch1-knockdown plasmid negative control–treated PVECs; Ko, Notch1-knockdown plasmid–treated PVECs.

To determine the direct impact of Notch1-DLL4 interaction on ZBP1 expression, we revealed that N1ICD and the Notch1-targeting transcription factors HES1 and HEY1 bind to the ZBP1 promoter, demonstrating that DLL4-Notch1 signaling positively regulates ZBP1 expression in lung endothelial cells (Supplemental Figure 3, A–C, and Supplemental Table 1). We next attempted to discover and develop a specific inhibitor of Notch1-DLL4. By using computational modeling, we identified 5 peptides derived from Notch1 extracellular domain, each 15–20 amino acids in length, as possible Notch1-DLL4 inhibitors. The identified peptides’ sequences based on Notch1-DLL4 interaction interface were DVDECALGANPCEHAGKCLN (411 to 431), CREGFSGPNCQTNINECA (779 to 796), GTYKCTCPQGYTGLNCQNLV (1,041 to 1,060), WCDSAPCKNGGRCWQTNTQY (1,062 to 1,081), and ECAGSPCHNGGTCEDGI (681 to 697) (Figure 4A and Supplemental Tables 2 and 3). To assess the inhibitory effects of 5 peptides (peptides 1, 2, 3, 4, and 5) on ZBP1 expression in pulmonary endothelial cells, PVECs were cocultured with DLL4^+^ neutrophils before being treated with varying concentrations of each peptide. Coculture with DLL4^+^ neutrophils significantly increased ZBP1 expression in PVECs. In contrast, treatment with peptide 5 significantly reduced ZBP1 expression, while peptides 1–4 had no significant effect on ZBP1 mRNA levels (Supplemental Figure 4). Peptide 5 was designed as the Notch1-DLL4 inhibitor (NDI); dose-response experiments revealed 10 μM NDI to be the effective concentration for ZBP1 inhibition, which was subsequently used for all in vitro studies. Computational modeling predicted the strong binding of NDI to DLL4 (Figure 4B). BIAcore data further confirmed the strong binding affinity between DLL4 and Notch1 with *K_D_* of 4.26 × 10^–8^ M (Figure 4C). Importantly, NDI treatment significantly disrupted Notch1-DLL4 interaction, increasing the *K_D_* by more than one order to 2.37 × 10^–7^ M (Figure 4D), indicating a weaker Notch1 and DLL4 binding in the presence of the inhibitor NDI. Thus, NDI was a new and specific Notch1-DLL4 inhibitor.

To determine the effect of NDI on DLL4^+^ neutrophil–induced pulmonary endothelial cell death, we cocultured PVECs with DLL4^+^ neutrophils or DLL4^–^ neutrophils in the presence or absence of NDI. To specifically target the Notch1 signaling pathway, we successfully knocked down Notch1 expression in PVECs by approximately 75% using a double-nickase plasmid transfection system. These Notch1-deficient PVECs and their corresponding negative control–transfected cells were then cocultured with DLL4^+^ neutrophils. Coculture with DLL4^+^ neutrophils significantly increased ZBP1 mRNA and protein expression in PVECs, and this was significantly attenuated by NDI treatment; moreover, ZBP1 expression was significantly decreased in Notch1-deficient PVECs (Figure 4, E and F). Similarly, DLL4^+^ neutrophils increased the levels of c-GSDMD, c-caspase-3, and p-MLKL in PVECs, while NDI and Notch1-deficient PVECs significantly reduced these markers of PANoptosis (Figure 4, G–I). DLL4^–^ neutrophils were not able to induce an increase of ZBP1, c-GSDMD, c-caspase-3, and p-MLKL in PVECs; therefore, NDI had less effect on the DLL4^–^ neutrophil coculture group. These results strongly support that DLL4^+^ neutrophils induce ZBP1 expression in PVECs via Notch1 signaling. NDI effectively attenuates DLL4^+^ neutrophil–induced PANoptosis in PVECs.

### NDI decreases DLL4^+^ neutrophil–induced pulmonary endothelial cell PANoptosis in murine CLP.

**Figure 5:**
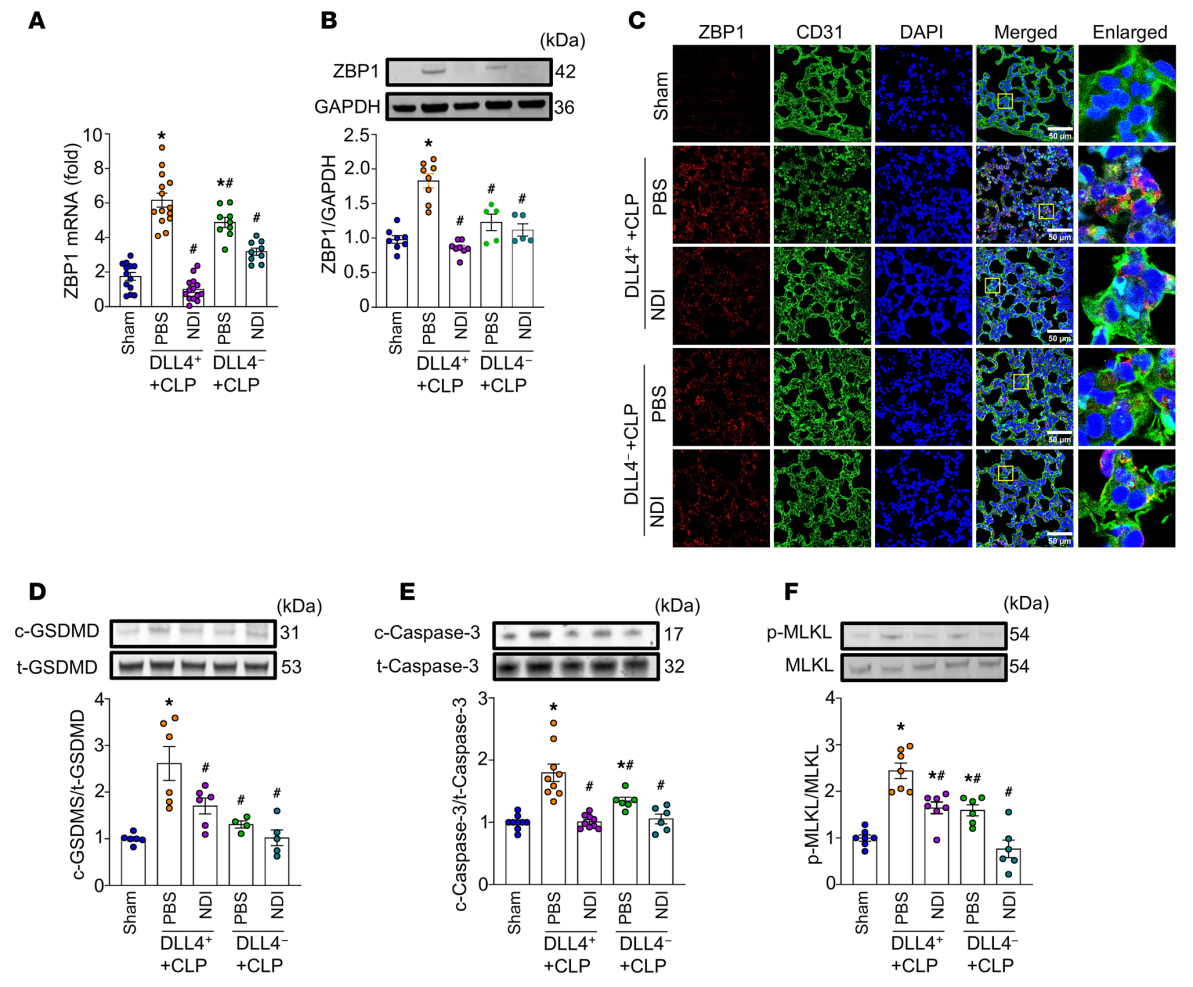
NDI attenuates PVEC PANoptosis by inhibiting Notch1-DLL4 interaction in murine CLP model. FACS-isolated DLL4^+^ neutrophils (1 × 10^6^/mL) or DLL4^–^ neutrophils (1 × 10^6^/mL) were retro-orbitally injected into mice at the time of CLP; then mice were treated with PBS or 10 mg/kg NDI. After 20 hours, lungs were harvested. (**A**) Total RNA was extracted from lung, and ZBP1 expression was analyzed by RT-PCR. Experiments were performed at least 3 times, and all data were analyzed. Data are expressed as means ± SEM and were analyzed using 1-way ANOVA. *n* = 9–14 per group. (**B** and **D**–**F**) Western blot analysis was performed to measure protein levels of ZBP1, c-GSDMD, t-GSDMD, c-caspase-3, t-caspase-3, p-MLKL, and MLKL in total lung tissue. Pyroptosis, apoptosis, and necroptosis were quantified as c/t GSDMD, c/t caspase-3, and p-MLKL/MLKL. Experiments were performed at least 3 times, and all data were analyzed. Data are expressed as means ± SEM and were analyzed using 1-way ANOVA. *n* = 5–9 per group. (**C**) Confocal microscopy was used to assess ZBP1 expression in lungs. ZBP1 was stained red, pulmonary endothelial cells were stained green (CD31), and nuclei were stained blue (DAPI). Scale bars: 50 μm. *P < 0.05 vs. sham. ^#^*P* < 0.05 vs. DLL4^+^ neutrophils–CLP/PBS.

We further investigated whether NDI could mitigate pulmonary endothelial PANoptosis induced by DLL4^+^ neutrophils in sepsis. We found that, compared with the sham and DLL4^–^ neutrophils/sepsis groups, the DLL4^+^ neutrophils/sepsis group had significantly higher ZBP1 mRNA and protein expression, whereas NDI effectively decreased ZBP1 expression (Figure 5, A and B). Immunohistochemical staining further confirmed that NDI reduced ZBP1 expression in pulmonary endothelial cells in sepsis (Figure 5C). NDI not only reduced ZBP1 expression but also attenuated the expression/activation of c-GSDMD, c-caspase-3, and p-MLKL (Figure 5, D–F). These results indicate that NDI effectively attenuated DLL4^+^ neutrophil–induced pulmonary endothelial cell PANoptosis during sepsis.

### NDI attenuates ALI and improves survival in sepsis.

**Figure 6:**
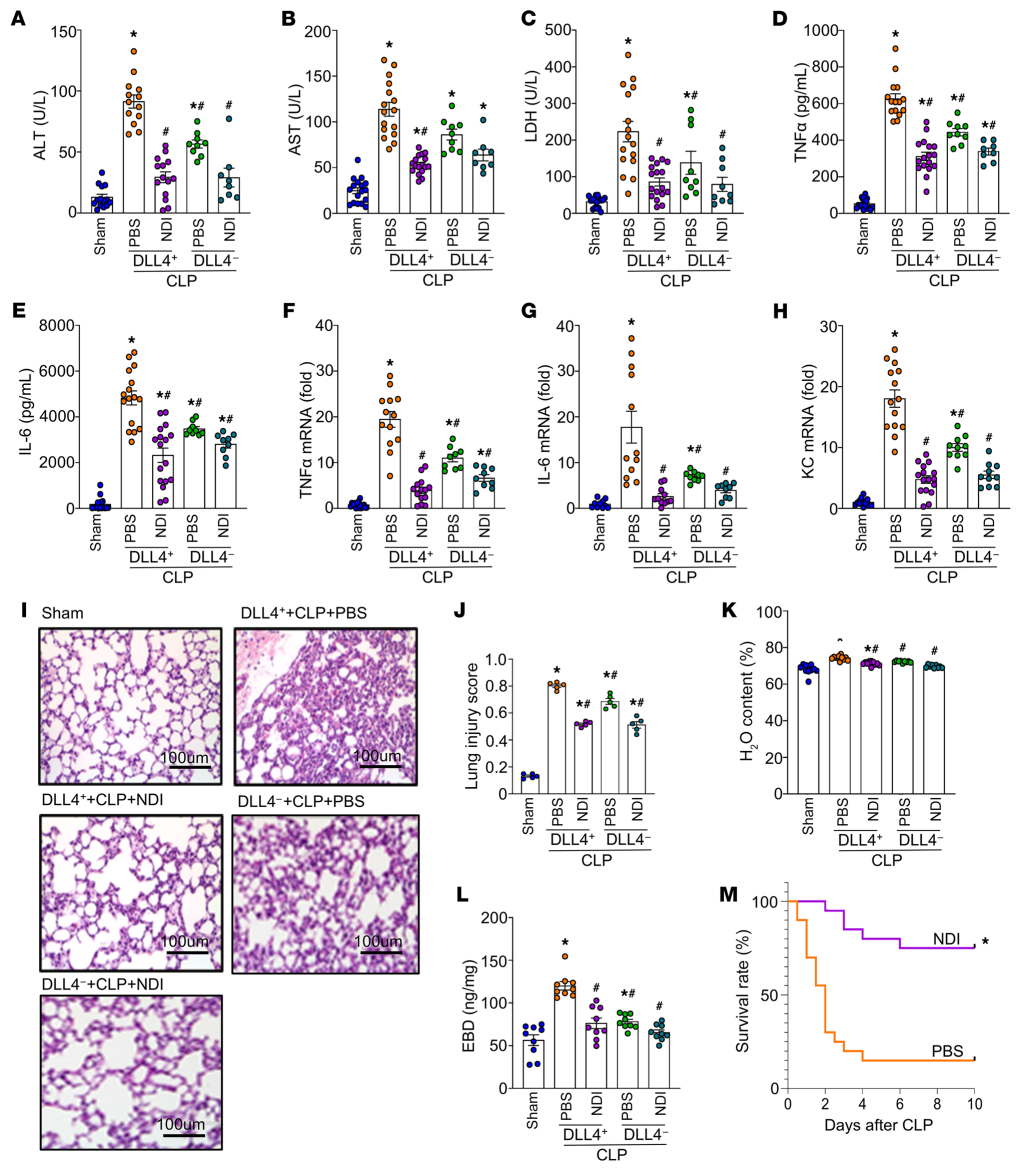
NDI attenuates ALI and inflammation and improves survival in sepsis. FACS-sorted DLL4^+^ neutrophils (1 × 10^6^/mL) or DLL4^–^ neutrophils (1 × 10^6^/mL) were retro-orbitally injected into mice at the time of CLP. C57BL/6 male mice were then treated with PBS or 10 mg/kg NDI. After 20 hours, lungs and blood were harvested. (**A**–**C**) Alanine aminotransferase (ALT), aspartate aminotransferase (AST), and lactate dehydrogenase (LDH) were determined using specific colorimetric enzymatic assays. (**D** and **E**) Serum levels of IL-6 and TNF-α were assessed by ELISA. (**F**–**H**) Lung mRNA levels of TNF-α, IL-6, and keratinocyte chemoattractant (KC) were assessed by PT-PCR. *n* = 8–17 per group. Data are representative of 3 independent experiments. Data are expressed as means ± SEM and were analyzed using 1-way ANOVA. *P < 0.05 vs. sham. ^#^*P* < 0.05 vs. DLL4^+^ neutrophils–CLP/PBS. (**I** and **J**) Lung tissue sections were stained with H&E to assess lung injury severity. Lung injury score (original magnification, ×200) is shown as a bar graph. *n* = 5 high-power fields per group. Scale bars: 100 μm. (**K** and **L**) Lung water content [(wet weight – dry weight)/wet weight %] and Evans blue dye (EBD) (EBD/dry lung tissue) were measured to evaluate pulmonary permeability. *n* = 9–12 per group. Experiments were performed at least 3 times, and all data were analyzed. Data are expressed as means ± SEM and were analyzed using 1-way ANOVA. *P < 0.05 vs. sham. ^#^*P* < 0.05 vs. DLL4^+^ neutrophils–CLP. C57BL/6 male mice were treated with PBS or 10 mg/kg NDI at the time of CLP. (**M**) Kaplan-Meier 10-day survival curve generated from PBS- and NDI-treated CLP mice. To better mimic the clinical scenario and assess the therapeutic potential of NDI, exogenous DLL4^+^ neutrophils were not used in the survival experiment. *N* = 20 mice per group. *P < 0.05 vs. vehicle (PBS).

Given that excessive PANoptosis can lead to a robust inflammatory response, our results showed that compared with the sham group and the DLL4^–^ neutrophil–injected CLP group, DLL4^+^ neutrophil–injected septic mice exhibited significantly higher serum levels of tissue injury markers (alanine aminotransferase, aspartate aminotransferase, and lactate dehydrogenase) (Figure 6, A–C) and proinflammatory cytokines (TNF-α and IL-6) (Figure 6, D and E). Additionally, lung tissue analysis revealed a significant increase in the mRNA expression of TNF-α, IL-6, and keratinocyte chemoattractant (KC) in comparison with the sham group (Figure 6, F–H). However, treatment with NDI significantly reduced the serum levels of hepatic and cell injury markers and proinflammatory cytokines caused by the DLL4^+^ neutrophils. Moreover, NDI significantly decreased the mRNA expression of TNF-α, IL-6, and KC in lung tissues (Figure 6, A–H). We further assessed lung injury severity by examining H&E-stained lung tissue sections. The lungs of DLL4^+^ neutrophil–injected septic mice exhibited increased alveolar congestion, proteinaceous debris, interstitial and alveolar neutrophil infiltration, intra-alveolar capillary hemorrhages, and damage to epithelial architecture. By contrast, NDI treatment effectively reduced lung injury, resulting in significantly lower lung injury scores (Figure 6, I and J). To assess the impact of NDI on pulmonary permeability, we measured lung water content and Evans blue dye extravasation in septic mice receiving DLL4^+^ neutrophils or DLL4^–^ neutrophils. Septic mice injected with DLL4^+^ neutrophils exhibited increased pulmonary permeability, evidenced by higher lung water content and Evans blue dye content compared with the sham group. This increase suggests endothelial dysfunction. By contrast, treatment with NDI significantly attenuated these effects, reducing both lung water content and Evans blue dye content in comparison with mice receiving DLL4^+^ neutrophils without NDI (Figure 6, K and L). These findings demonstrated that NDI treatment significantly attenuates DLL4^+^ neutrophil–induced ALI during sepsis. Importantly, NDI treatment led to a significant improvement in survival rates of septic mice compared with the PBS-injected septic mice from 15% to 75% (Figure 6M). These findings indicated that NDI confers significant protection during sepsis by attenuating systemic inflammation and injury markers, decreasing ALI, and improving survival.

## Discussion

**Figure 7:**
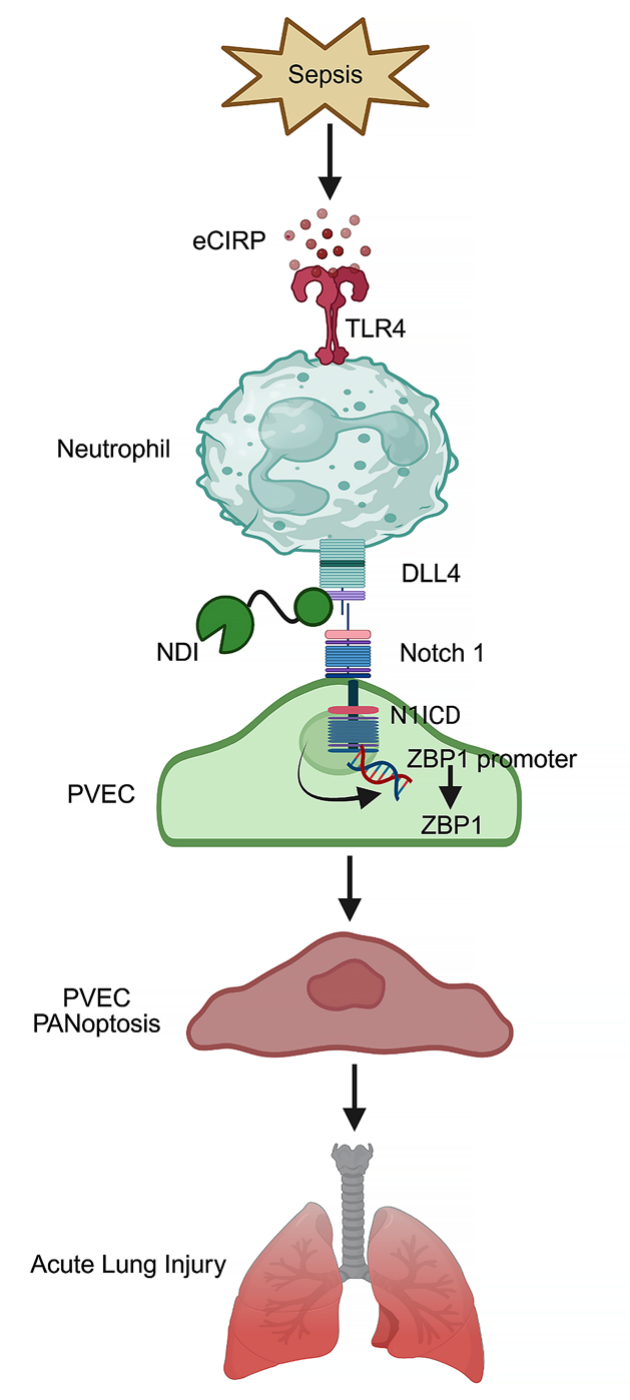
Schema of findings. DLL4^+^ neutrophils induce pulmonary endothelial cell PANoptosis via the Notch1-DLL4 pathway in sepsis. eCIRP induces neutrophils to express DLL4, and these DLL4^+^ neutrophils interact with PVECs through the Notch1-DLL4 pathway. This interaction triggers endothelial PANoptosis, driving inflammation and ALI in sepsis. NDI effectively inhibits the Notch1-DLL4 interaction, reducing endothelial PANoptosis and ALI, thereby improving survival outcomes in sepsis. The schema was created in BioRender (Murao A, 2025, https://BioRender.com/y914a2i).

In this study, we have identified a distinct DLL4^+^ neutrophil phenotype that promotes ALI in sepsis. Our results demonstrate that eCIRP, a new DAMP, promotes DLL4^+^ neutrophils, which induce lung endothelial cell PANoptosis via the Notch1-DLL4 pathway, exacerbating ALI and increasing mortality in sepsis. Importantly, we have developed NDI, a potentially novel antagonist peptide that effectively blocks the Notch1-DLL4 interaction, reducing endothelial PANoptosis, systemic inflammation, ALI, and mortality in sepsis (Figure 7). Thus, our study reveals what we believe to be a novel pathophysiology of ALI and identifies a therapeutic target for mitigating sepsis-induced ALI.

Notch signaling is vital in processes like cell differentiation, proliferation, and apoptosis. DLL4, expressed primarily in myeloid cells, including macrophages and dendritic cells, plays a key role in modulating adaptive immune responses through its interaction with the Notch1 signaling pathway (27, 28). To evaluate expression of different Notch ligands in neutrophils under septic conditions, we analyzed RNA sequencing data from septic mice and found that DLL4 was the most highly expressed ligand (Supplemental Figure 5, A and B). By analyzing our archived single-cell RNA sequencing data, we found that DLL4 is highly expressed in eCIRP-activated neutrophils (29). Furthermore, we treated bone marrow–derived neutrophils with eCIRP, and assessed expression of DLL1, DLL3, and DLL4 by reverse transcriptase PCR (RT-PCR). The results showed that eCIRP stimulation selectively upregulated DLL4, with no significant induction of DLL1 or DLL3 (Supplemental Figure 5C). Importantly, DLL4 has a well-established high binding affinity to Notch1, which supports our focus on the DLL4-Notch1 signaling axis (30). While DLL4 expression has been studied in different immune or non-immune cells (30–32), its expression in neutrophils has remained unclear. To our knowledge, our study is the first to show that neutrophils express DLL4 both transcriptionally and translationally. Data from TLR4^–/–^ mice highlight the role of eCIRP in driving DLL4 expression via TLR4 signaling. Excessive neutrophil activation is hallmark in sepsis-induced tissue damage and organ dysfunction (33), highlighting the significance of targeting harmful neutrophil subpopulations such as DLL4^+^ neutrophils to mitigate ALI. Our findings show not only that eCIRP induces the production of DLL4^+^ neutrophils but also that these cells contribute to inflammation, ALI, and mortality. During sepsis, eCIRP-induced DLL4^+^ neutrophils exist in blood circulation and organs, such as lungs. The identification of the role of the Notch1-DLL4 axis, facilitated by neutrophil–endothelial cell crosstalk within the pulmonary microenvironment, is crucial for understanding the development of ALI and positions DLL4-expressing neutrophils as a potential therapeutic target in sepsis-induced ALI. While TLR4 can be activated by eCIRP, it is also responsive to other DAMPs, such as HMGB1. Therefore, DLL4^+^ neutrophils might be activated by DAMPs other than eCIRP.

To further investigate the role of DLL4^+^ neutrophils in ALI, we examined how these cells influence PVECs. During inflammation, neutrophils accumulate in the lungs (34), interact with vascular endothelial cells, and disrupt endothelial function (35). Furthermore, our data demonstrate that neutrophils successfully migrate to the lungs with over 90% viability, where DLL4^+^ neutrophils directly contact pulmonary endothelial cells and induce endothelial cell injury. Given the elevated Notch1 expression in inflamed PVECs (36), our in silico model showed that DLL4 strongly binds with Notch1, and that Notch1-DLL4 signaling provides a pathway for neutrophil-PVEC communication. PANoptosis, a recently characterized form of programmed cell death, integrates pyroptosis, apoptosis, and necroptosis (37). While physiological cell death maintains homeostasis, dysregulated PANoptosis can trigger excessive inflammation and tissue damage, hallmarks of ALI. Given that pathogens implicated in ALI (e.g., influenza A virus, SARS-CoV-2) are known to induce PANoptosis, particularly in endothelial cells, it is plausible that endothelial cell PANoptosis represents a significant contributor to ALI pathogenesis (17, 21, 38). Our data demonstrate that DLL4^+^ neutrophils induce ZBP1-mediated PANoptosis in lung endothelial cells. This result revealed that, during inflammation, neutrophils not only express DLL4, but also communicate with PVECs via the Notch1-DLL4 pathway, leading to endothelial barrier damage. Originally described in macrophages, epithelial cells, and endothelial cells, PANoptosis is increasingly recognized as an important inflammatory process in sepsis, sterile inflammation, and cancer (20, 39). PANoptosomes are formed by cytosolic pattern recognition receptors in response to pathogens such as viruses, pathogen-associated molecular patterns, DAMPs, or the cytokines produced downstream (40). In the context of SARS-CoV-2 infection, combined treatment with TNF-α and IFN-γ activates the JAK/STAT1/IRF1 axis, inducing nitric oxide production and driving caspase-8/FADD–mediated PANoptosis (21). While previous studies have primarily focused on pathogen-induced PANoptosis (37), our findings reveal a mechanism by which DLL4^+^ neutrophils directly induce PANoptosis through a cell-cell contact–dependent Notch1-DLL4 pathway. This highlights the role of immune cell–driven mechanisms in sepsis-induced organ dysfunction. Moreover, this discovery reveals the impact of cellular crosstalk in driving distinct cell death modalities, specifically PANoptosis. Our data demonstrate that knockdown of Notch1 expression in endothelial cells markedly attenuates PANoptosis, indicating that Notch1 plays a critical role in endothelial cell PANoptosis. These findings suggest that targeting the Notch1-DLL4 pathway may represent a promising therapeutic strategy to mitigate endothelial injury and sepsis-induced ALI.

To counteract the detrimental effects of DLL4^+^ neutrophils, we designed Notch1-derived peptides to competitively inhibit Notch1-DLL4 binding. This targeted approach offers greater specificity than DAPT, which broadly blocks Notch1 activation. Furthermore, because DAMPs other than eCIRP can also induce DLL4 expression in neutrophils, an anti-eCIRP strategy alone may not be fully effective. A specific Notch1-DLL4 inhibitor could potentially address this limitation by inhibiting the activation of DLL4^+^ neutrophils induced by these other DAMPs. Among the tested peptides, NDI most effectively inhibited DLL4^+^ neutrophil–induced PANoptosis in PVECs, as demonstrated by reduced ZBP1 expression. Furthermore, NDI’s solubility in PBS enhances its therapeutic potential by obviating the need for toxic solvents. A scrambled peptide served as an in vitro control to account for any effects attributable to the NDI backbone. Only male mice were used to avoid any sex-dependent hormonal influences on the outcome of sepsis (41).

Computational modeling was used to explore the mechanisms by which the Notch1-DLL4 pathway promotes ZBP1-mediated PANoptosis. We found that Notch1 activation induces ZBP1 expression via 2 pathways: (a) upon DLL4 binding, the N1ICD is activated, directly promoting ZBP1 transcription; and (b) N1ICD activates downstream Notch1 target genes, including HEY1 and HES1, which also enhance ZBP1 transcription. These findings reveal that Notch1-DLL4 binding promotes ZBP1-mediated PANoptosis through transcriptional regulation of ZBP1 mediated by both N1ICD and Notch1 target genes. Previous study shows that ZBP1 deficiency protects against organ injury and improves survival in the CLP sepsis model. This supports the pathogenic role of ZBP1 in sepsis-induced inflammation and organ failure (42).

Inhibiting Notch1-DLL4 signaling reduces N1ICD activation and Notch1 target gene expression, thereby potentially decreasing ZBP1 expression. Although N1ICD activation is essential for vascular development and maintenance at the embryonic stage (43), our study showed that NDI improved vascular barrier function and reduced pulmonary permeability, decreased inflammatory factors, and attenuated mortality in septic mice, thereby supporting its therapeutic potential in the pathophysiological conditions. To better mimic the clinical scenario and assess the therapeutic potential of NDI, in the survival experiment, we chose not to artificially introduce additional DLL4^+^ neutrophils. CLP alone induces approximately 75% mortality. Since DLL4^+^ neutrophils exacerbate organ injury and promote PANoptosis, administering additional DLL4^+^ neutrophils would likely result in near-universal lethality, precluding meaningful assessment of NDI’s survival benefit. In the survival experiment, treatment with NDI reduced CLP-induced mortality from 75% to 15%. This striking improvement underscores the critical role of this pathway in sepsis-induced lethality and highlights its potential as a therapeutic target even without DLL4^+^ PMN infusion in sepsis. In this study, we selected an in vivo dosage of 10 mg/kg for NDI treatment. This dose was chosen primarily on the basis of our laboratory’s prior experience; we have consistently used this dose in mouse CLP models with reproducible efficacy and without observable toxicity (39, 44). It is important to note that in vitro concentration (10 μM) cannot be directly extrapolated to in vivo dosing because of differences in pharmacokinetics, tissue distribution, and metabolic clearance. Therefore, we adopted 10 mg/kg as a pragmatic and experimentally validated regimen that ensures sufficient systemic exposure, while also maintaining consistency with our laboratory’s established protocols and enabling direct comparison across different peptide studies. The findings observed with NDI warrant further investigation of its pharmacokinetics, toxicity, and potential off-target effects. However, the lack of observed adverse effects in treated animals, coupled with the observed survival benefit, supports its continued development as a therapeutic candidate. Future studies will explore additional characteristics of DLL4^+^ neutrophils, including their aging, migration patterns, NET formation, and ROS production, beyond their role in ZBP1-mediated PANoptosis. In this study, we focused on septic conditions, in which systemic inflammation promotes neutrophil recruitment to the lungs via the bloodstream. Given that infiltrating neutrophils primarily interact with pulmonary endothelial cells, we investigated whether DLL4^+^ neutrophils induce PANoptosis in pulmonary endothelial cells under septic conditions. While we have not yet evaluated the effects of DLL4^+^ neutrophils in non-septic models, we plan to expand our investigation into other cell types that express Notch1 and disease models (e.g., ischemia/reperfusion injury, hemorrhagic shock) in future studies.

In summary, our study is the first to identify DLL4^+^ neutrophils as a distinct neutrophil phenotype during sepsis. These DLL4^+^ neutrophils interact with PVECs via the Notch1-DLL4 pathway to promote endothelial PANoptosis, leading to the development of ALI. Furthermore, we developed NDI, a peptide that blocks Notch1-DLL4 interaction, mitigating endothelial PANoptosis, inflammation, and ALI and significantly improving survival in a murine model of sepsis. These findings provide valuable insights into the mechanisms of sepsis-induced ALI and highlight DLL4^+^ neutrophils as a promising therapeutic target for sepsis-induced ALI.

## Methods

### Sex as a biological variable.

Only male mice were used in this study to exclude the sex-specific differences in sepsis. It is unknown whether the findings are relevant to female mice.

### Recombinant proteins and peptides.

Recombinant murine (rm) DLL4 and rmNotch1 proteins were purchased from R&D Systems (catalog 1389-D4-050/CF and 5267-TK). rmCIRP (denoted as eCIRP) was produced in our laboratory (11). The Notch1-DLL4 interaction inhibitor peptides (peptides 1–5) were designed using the amino acid sequences present in the Notch1 extracellular domain that specifically interacts with DLL4 (protein-protein interaction interface and the structural similarity between the Notch1 extracellular domain and the DLL4). The binding affinity of peptides was estimated by measurement of the interaction between DLL4 and peptides. The peptides were docked into the DLL4, and the thermodynamic properties of interaction between DLL4 and peptides were calculated. The binding energies (Δ^i^G) of peptides were –13.0, –14.8, –19.7, –20.8, and –15.0 in kcal/mol. All peptides were synthesized by GenScript.

### BIAcore assay.

To determine rmDLL4’s direct interaction with rmNotch1, surface plasmon resonance (OpenSPR, Nicoya) was performed. High-sensitivity nitrilotriacetic acid (HS-NTA) sensors were used. Binding reactions were performed in running buffer (PBS, 0.005% P20, pH 7.4). The HS-NTA sensor was first cleaned by injection with 10 mM HCl (150 μL) followed by injection of 150 μL of 350 mM EDTA. Then the surface was activated by an injection of 150 μL of 40 mM NiCl_2_. rmNotch1 was diluted to 25 μg/mL with the 10 mM acetate buffer (pH 5) and immobilized to channel 2. rmDLL4 was injected as an analyte at concentrations of 5 nM, 12.5 nM, 50 nM, and 100 nM with a flow rate of 40 μL/min. For the effect of NDI efficacy on rmDLL4 binding to rmNotch1, the same HS-NTA sensor was used. rmDLL4 at all the concentrations used for binding with rmNotch1 was preincubated with 1 μg/mL NDI for 30 minutes at room temperature, and then the complexes were injected to channels 1 and 2. Binding reactions were carried out with the same running buffer at a flow rate of 40 μL/min at 20°C. Channel 1 was used as a control to evaluate nonspecific binding. The real-time interaction data were analyzed by Trace Drawer (Nicoya). The signals from channel 1 were subtracted from channel 2 coated with the ligand for all samples. Data were globally fitted for 1:1 binding (one-to-one model).

### Endothelial cell culture and Notch1 knockdown.

Immortalized mouse (*Mus musculus*) lung endothelial cells with polyoma middle T (PVECs) were purchased from Applied Biological Materials Inc. (ABM) and grown in 1:1 PriGrow VI Medium and PriGrow IV Medium (ABM).

PVECs were transfected with a double-nickase plasmid targeting Notch1 using the UltraCruz transfection reagent (Santa Cruz Biotechnology), according to the manufacturer’s protocol. Briefly, PVECs were seeded in 6-well plates at a density of 5 × 10^5^ cells per well in 3 mL of antibiotic-free growth medium and cultured for 24 hours. For transfection, 4 μg of either Notch1-specific double-nickase plasmid or a negative control plasmid was diluted in 150 μL plasmid transfection medium (solution A) and combined with 15 μL UltraCruz transfection reagent diluted in 150 μL plasmid transfection medium (solution B). The 2 solutions were gently mixed, incubated briefly at room temperature for 20 minutes, and added dropwise to the cells. After 24 hours of incubation, Notch1 mRNA expression was assessed by RT-PCR to confirm knockdown efficiency.

### Human neutrophils.

Blood samples were obtained from the deidentified healthy human subjects. Written informed consent was obtained from all participants, and human subject protocols were approved by the Institutional Review Board of the North Shore University Hospital. Five milliliters of blood was collected by venipuncture and placed in EDTA blood collection tubes. We isolated the neutrophils from fresh blood samples by immunomagnetic cell separation using an EasySep Direct Human Neutrophil Isolation Kit (catalog 19666, Stem Cell Technologies). A total of 20 × 10^6^ neutrophils were obtained from 5 mL of blood. Neutrophils (1 × 10^6^ cells/mL) were stimulated with 1 μg/mL eCIRP for 12 hours. The cells were then stained with anti-CD66b (clone G10F5, catalog 984102, BioLegend) and DLL4 antibodies (clone MHD4-46, catalog 346506, BioLegend), and the frequencies (percentage) of DLL4^+^ neutrophils were detected by flow cytometry.

### Experimental animals.

Male 8-week-old C57BL/6 mice were purchased from Charles River Laboratories. TLR4^–/–^ mice were obtained from Kevin Tracey (Feinstein Institutes for Medical Research). Mice were housed at approximately 23°C with a 12-hour light/12-hour dark cycle and given standard laboratory food and water ad libitum. All experiments were performed following the NIH guidelines for experimental animals. The animal protocol was approved by the Feinstein Institutes’ Institutional Animal Care and Use Committee. Animals were randomly assigned to the sham, vehicle, or adoptive transfer groups.

### Animal model of polymicrobial sepsis.

Sepsis was induced in mice by cecal ligation and puncture (CLP) as described previously (45, 46). Mice were anesthetized with isoflurane to a surgical plane and placed in a supine position. CLP was performed through a midline laparotomy. The abdomen was shaved and disinfected. Through a 2 cm midline incision, the cecum was exposed and then ligated with a 4-0 silk suture 1 cm proximal from the distal cecal extremity. For 20-hour CLP experiments, the cecum was punctured twice with a 22-gauge needle. A small amount of cecal content was extruded through both holes, and the ligated cecum was returned to the peritoneal cavity. The wound was closed in layers. For 10-day sepsis survival studies, the cecum was punctured once with a 22-gauge needle. A small amount of cecal content was extruded. Mice were allocated to the vehicle and adoptive transfer of DLL4^+^ neutrophils or DLL4^–^ neutrophils groups. Adoptive transfer mice received a retro-orbital injection of 1 × 10^6^ cells per mouse DLL4^+^ neutrophils or DLL4^–^ neutrophils in 100 mL phosphate-buffered saline (PBS) immediately after abdominal closure in both the 20-hour experiments and the survival study. Vehicle groups received an equivalent volume of PBS, while treatment groups received NDI 10 mg/kg in 100 mL PBS via retro-orbital injection after CLP. This dose was chosen primarily on the basis of our laboratory’s prior experience, we have consistently used this dose in mouse CLP models with reproducible efficacy and without observable toxicity (39, 44). Sham-operated animals underwent a laparotomy without CLP. After closure, the mice received a subcutaneous injection of 0.5 mL of normal saline to overcome surgery-induced dehydration. For 20-hour experiments, animals were not injected with antibiotics; however, for survival studies, animals were given 0.5 mg per kg body weight imipenem (lot 4D21K05, WG Critical Care LLC) subcutaneously in 0.5 mL saline once at the end of CLP. All mice were subcutaneously injected with a single dose of 0.1 mg/kg buprenorphine after CLP.

### Isolation and purification of bone marrow–derived neutrophils.

Mice were anesthetized by 2% isoflurane inhalation, and the femora and tibiae were dissected. The bone marrow was flushed out with calcium- and magnesium-free HBSS using a 25-gauge needle into a Petri dish. Suspensions of cells were filtered through a 70 μm cell strainer (Corning), and bone marrow–derived neutrophils (BMDNs) were purified by negative selection using the EasySep mouse neutrophil enrichment kit (catalog 19762, STEMCELL Technologies). The purity of the sorted neutrophils was assessed by staining of the cells with APC-Ly6G antibody (clone 1A8, BioLegend) using a BD LSRFortessa flow cytometer (BD Biosciences).

### Detection and flow sorting of DLL4^+^ neutrophils and DLL4^–^ neutrophils.

In the time course experiments, BMDNs (1 × 10^6^) were stimulated with eCIRP (1 μg/mL) for 2, 4, 6, and 12 hours. In the dose-response experiments, BMDNs (1 × 10^6^) were treated with 0, 0.2, 0.5, or 1.0 μg/mL eCIRP for 12 hours. Single-cell suspensions were then stained with APC-conjugated anti–mouse Ly6G (clone 1A8, catalog 127614, BioLegend) and PE-conjugated anti-DLL4 (clone HMD4-1, catalog 130807, BioLegend), and analyzed by flow cytometry (BD LSRFortessa, BD Biosciences) to detect DLL4^+^ neutrophils (Ly6G^+^DLL4^+^). Unstained cells were used to set voltages, and single-color controls were applied for compensation adjustment. Two hundred fifty microliters of whole blood obtained from sham, CLP, and PBS- or NDI-treated mice was taken into Falcon 15 mL conical tubes, and 5 mL red blood cell (RBC) lysis buffer (BD Biosciences) was added. After 1–2 minutes of incubation at room temperature, the samples were centrifuged at 300*g* for 10 minutes. Supernatants were aspirated, and the cell pellet was washed by suspension of the cells in 5 mL fluorescence-activated cell sorting (FACS) buffer containing 2% fetal bovine serum and centrifuged at 300*g* for 10 minutes. The supernatant was discarded, and the cell pellet was dissolved in 500 mL FACS buffer. Lung tissues were finely diced using a sterile surgical blade and suspended in Ca^++^- and Mg^++^-free Hanks balanced salt solution (HBSS; Corning). Tissue digestion was performed in HBSS containing 100 U/mL of collagenase I (Worthington Biochemical) and 20 U/mL DNase I (Worthington Biochemical) at 37°C for 30 minutes with periodic shaking. Digested tissue fragments were crushed with a 10 mL syringe plunger and passed through a 70 μm cell strainer (Corning). Lysis of RBCs in lung cell suspensions was conducted using RBC lysis buffer (BD Biosciences). The isolated lung cells were counted using a microscope (Eclipse TS100, Nikon). To detect mouse DLL4^+^ neutrophils, single-cell suspensions were stained with anti-mouse APC-Ly6G (clone 1A8, catalog 127614, BioLegend) and PE-DLL4 (clone HMD4-1, catalog 130807, BioLegend) and assessed for the detection of DLL4^+^ neutrophils (Ly6G^+^DLL4^+^) by flow cytometry (BD LSRFortessa, BD Biosciences). Unstained cells were used to establish the flow cytometer voltage setting, and single-color positive controls were used to adjust the compensation. The acquisition was performed on 50,000 events using a BD LSRFortessa flow cytometer (BD Biosciences), and data were analyzed with FlowJo software (Tree Star). BMDNs were stimulated with eCIRP (1 mg/mL) for 2 hours. This time point was selected to maximize the viability and functionality of DLL4^+^ neutrophils for downstream experiments, given their relatively short lifespan. After stimulation, cells were washed with PBS and resuspended in 1 mL FACS buffer, and DLL4^+^ neutrophils (Ly6G^+^DLL4^+^) and DLL4^–^ neutrophils (Ly6G^+^DLL4^–^) were sorted by flow cytometry using a BD FACSAria IIu cell sorter (BD Biosciences).

### Viability assessment of adoptively transferred DLL4^+^ and DLL4^–^ neutrophils.

BMDNs were isolated from wild-type mice and stimulated with eCIRP (1 μg/mL) for 2 hours. DLL4^+^ and DLL4^–^ neutrophils were subsequently sorted by FACS, then labeled with CFSE (catalog C34570, Thermo Fisher Scientific). CFSE-labeled DLL4^+^ and DLL4^–^ neutrophils (1 × 10^6^ cells in 100 μL per mouse) were delivered into mice by retro-orbital injection at the time of CLP. At 20 hours after CLP, lungs were harvested, single-cell suspensions were stained with live/dead cell staining (catalog C34557, Thermo Fisher Scientific), and CFSE^+^ neutrophils and their viability were assessed by flow cytometry.

### Flow sorting of CD31^+^ pulmonary endothelial cells.

Lungs were harvested 20 hours after CLP and processed into single-cell suspensions as described above. Cells were washed with PBS, resuspended in 1 mL of FACS buffer, and stained with PE–anti-CD31 antibody (clone W18222B, catalog 160203, BioLegend). CD31^+^ cells were sorted using a FACSAria IIu cell sorter (BD Biosciences). Purified CD31^+^ cells were subsequently analyzed for ZBP1 expression by quantitative PCR (qPCR) and Western blotting.

### DLL4^+^ neutrophil/DLL4^–^ neutrophil and PVEC cocultures.

Wild-type PVECs (5 × 10^5^) were cocultured with sorted DLL4^+^ neutrophils (5 × 10^5^) or DLL4^–^ neutrophils (5 × 10^5^) and then treated with different dosages of peptides or 10 μM DAPT (Thermo Fisher Scientific), 10 μM NDI (GenScript), or 10 μM NDI scramble (GenScript). Notch1-knockdown PVECs (5 × 10^5^) were cocultured with sorted DLL4^+^ neutrophils (5 × 10^5^). After 16 hours, neutrophils were washed away by PBS, and PVECs were collected for further experiments. Cell viability was evaluated by propidium iodide staining using the FITC Annexin V Apoptosis Detection Kit I (catalog 556547, BD Pharmingen). Cell death was quantified as the percentage of propidium iodide–positive cells by flow cytometry.

### Real-time qPCR.

mRNA was extracted from lung tissues with TRIzol reagent (Invitrogen, Thermo Fisher Scientific). An equal amount (3 mg) of mRNA was reverse-transcribed into cDNA using the reverse transcriptase enzyme (Invitrogen, Thermo Fisher Scientific). qPCR was performed from the diluted cDNA templates with forward and reverse primers (Supplemental Table 4) and SYBR Green PCR Master Mix (Applied Biosystems, Thermo Fisher Scientific) using an Applied Biosystems 7300 real-time PCR machine. Mouse β-actin served as an internal control gene for normalization. Relative expression of mRNA was represented as fold change in comparison with the sham group.

### Assessment of organ injury markers.

Serum levels of alanine aminotransferase, aspartate aminotransferase, and lactate dehydrogenase were determined using specific colorimetric enzymatic assays (Pointe Scientific Inc.) according to the manufacturer’s instructions.

### ELISA.

Blood was collected by heart puncture and centrifuged at 1,500*g* and 4°C for 10 minutes, and the plasma was separated to assess cytokine levels using enzyme-linked immunosorbent assay (ELISA) kits (BD Biosciences). The plasma TNF-α (catalog 558534) and IL-6 (catalog 555240) levels were measured by ELISA.

### Immunofluorescence assay for lung tissue sections.

For immunohistochemistry staining, paraffin-embedded lung tissue sections were deparaffinized and dewaxed in xylene and a series of ethanol. The sections were permeabilized using Triton X-100 and blocked with normal goat serum for 1 hour at room temperature. Subsequently, they were incubated overnight at 4°C with the diluted primary antibodies against ZBP1 (catalog PA5-20455, Thermo Fisher Scientific; 1:1,000) and CD31 (catalog 3528S, Cell Signaling Technology; 1:250). After incubation with appropriate secondary antibodies, the sections were counterstained with DAPI. The Vector TrueVIEW Autofluorescence Quenching Kit (catalog SP-8400-15, Vector Laboratories) was used to remove autofluorescence from the tissue sections before coverslips were mounted using ProLong Gold antifade reagent (catalog P36934, Thermo Fisher Scientific). The section was examined using a ZEISS LSM 900 confocal microscope. The images were analyzed with ImageJ Fiji (NIH).

### Western blot assays.

PVECs and lung tissue were collected in previous steps. Whole-cell proteins were extracted, and the protein concentration was measured using an ABC protein assay kit (catalog 5000002, Bio-Rad). Proteins were separated using NuPAGE 4%–12% Bis-Tris gels (Invitrogen), transferred to PVDF membranes, and immunoblotted with the antibodies indicated in the figure legends according to the manufacturer’s recommendations. The ZBP1 antibody (catalog PA5-20455; 1:1,000) used in this study was obtained from Thermo Fisher Scientific, and the c-GSDMD (catalog 10137S; 1:250), GSDMD (catalog 39754S; 1:1000), c-caspase-3 (catalog 9661L; 1:250), caspase-3 (catalog 9662S; 1:1,000), p-MLKL (catalog 37333S; 1:250), MLKL (catalog 37705S; 1:1,000), N1ICD (c-Notch1) (catalog 4147S; 1:250), and Notch1 (catalog 3608S; 1:1,000) antibodies were obtained from Cell Signaling Technology. GAPDH (catalog 60004-1-Ig; 1:10,000) and β-actin (catalog A5441, Sigma-Aldrich; 1:5,000) were obtained from ProteinTech. After the blots were incubated with primary antibodies, the membranes were washed 3 times, and the blots were subsequently incubated with the corresponding fluorescent secondary antibody (LI-COR). Bands were detected using an Odyssey FC Dual-Mode Imaging system 2800 (LI-COR).

### Histological examination.

Lung tissues were fixed in 10% formalin before being embedded in paraffin. Blocks were cut into 5 μm sections and stained with H&E. Slides were evaluated under light microscopy to assess the degree of lung injury. Scoring was done using a system created by the American Thoracic Society (47). Scores ranged from 0 to 1 and were based on proteinaceous debris in the airspaces, the degree of septal thickening, and neutrophil infiltration in the alveolar and interstitial spaces. The average score per field was calculated at ×200 original magnification.

### Lung extravasation assay.

Lung vascular leakage was quantified using the Evans blue dye–labeled (EBD-labeled) albumin extravasation assay (48). At 20 hours after CLP, mice were injected with EBD (20 mg/kg in 100 μL; Sigma-Aldrich) via retro-orbital injection. At 1 hour after EBD administration (4.5 hours after the end of resuscitation), the lungs were perfused with heparinized (1 U/mL) normal saline via right ventricle injection to remove intravascular dye. The whole lung was dehydrated for 48 hours at 60°C, immersed in formamide (1 mL/g wet weight; Sigma-Aldrich) at 37°C for 48 hours, and centrifuged at 5,000*g* for 30 minutes. The amount of extracted EBD in the supernatant was quantified by spectrophotometry. The concentration of extravasated EBD-albumin in lung homogenates was expressed as nanograms EBD per milligram of dry lung tissue. Lung water content was calculated as (wet weight – dry weight)/wet weight %.

### Assessment of DLL1, DLL3, and DLL4 expression in murine CLP model.

We analyzed a single-cell RNA sequencing dataset of spleen and peritoneal cells isolated from sham and CLP mice that we previously published (Gene Expression Omnibus GSE249975; ref. 49). Analysis was performed using the Cellenics platform (Biomage). Neutrophil clusters were annotated based on the ScType system, (https://github.com/IanevskiAleksandr/sc-type). and DLL1, DLL3, and DLL4 expression was evaluated.

BMDNs (1 × 10^6^) were treated with eCIRP (1 μg/mL) for 12 hours, and DLL1, DLL3, and DLL4 were analyzed by qPCR.

### Statistics.

Data represented in the figures are expressed as mean ± SEM and were compared by 2-tailed Student’s *t* test for 2 groups or 1-way ANOVA using Student-Newman-Keuls post hoc analysis for multiple groups. Survival rates were analyzed by the Kaplan-Meier estimator and compared using a log-rank test. Differences in values were considered significant if *P* was less than or equal to 0.05. Data analysis was carried out using GraphPad Prism graphing and statistical software (GraphPad Software).

### Study approval.

The present studies using live animals were reviewed and approved by the Institutional Animal Care and Use Committee of the Feinstein Institutes for Medical Research. Human cells were collected from consenting volunteer donors as part of Institutional Review Board (IRB) protocol 22-0458, reviewed and approved by the IRB of the Feinstein Institutes for Medical Research.

### Data availability.

All reported data values are available in the Supporting Data Values file.

## Author contributions

HJ and MA outlined the experiments. HJ, SH, JL, and AM performed in vitro and in vivo experiments. AJ did computational modeling. GM did BIAcore analysis. HJ and MA analyzed the data and wrote the manuscript. PW critically reviewed and edited the manuscript. MA and PW conceived the idea. PW and MA supervised the project.

## Funding support

This work is the result of NIH funding, in whole, and is subject to the NIH Public Access Policy. Through acceptance of this federal funding, the NIH has been given a right to make the work publicly available in PubMed Central.

- NIH grants R35GM118337, R01GM129633, and R01HL076179.

## Supplementary Material

Supplemental data

Unedited blot and gel images

Supporting data values

## Version 1 12/15/2025

Electronic publication
