# Supplementary material for: DLL4+ neutrophils promote Notch1-mediated endothelial PANoptosis to exacerbate acute lung injury in sepsis
Source: J Clin Invest. 2025 Dec 15;135(24):e194310. doi: 10.1172/JCI194310 (PMC12700550; doi:10.1172/JCI194310)
Supplement: Supplemental data [file jci-135-194310-s047.pdf]

Supplemental Figure 1

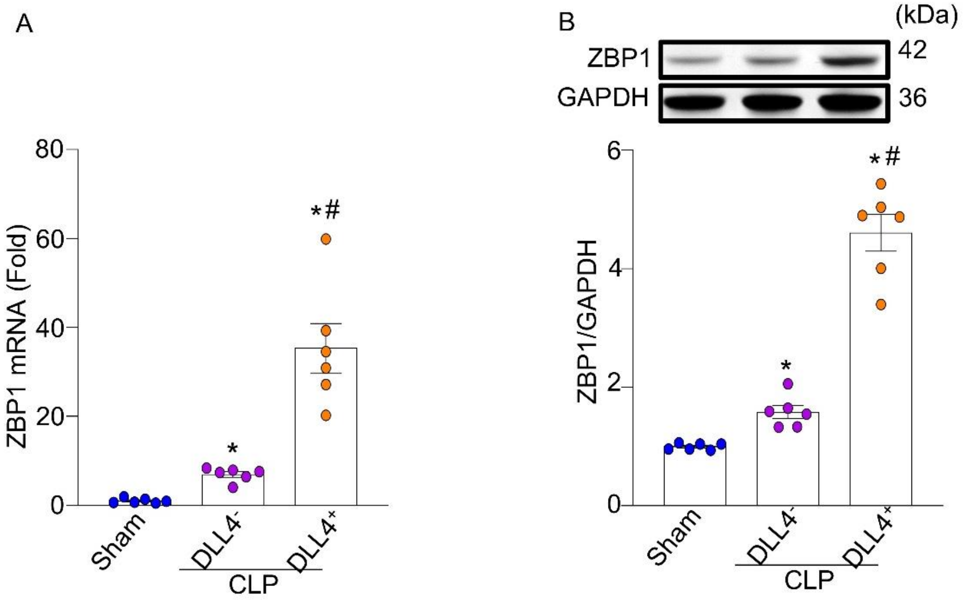

Supplemental Figure 1: DLL4$^+$ neutrophils promote ZBP1 in pulmonary endothelial cells in murine CLP model. BMDNs from WT mice were stimulated with eCIRP for 2 h. FACS was used to isolate DLL4$^+$ and DLL4$^-$ neutrophils, which were then injected via the retro-orbital route concurrently with CLP (1×10^6 cells/mouse). Lungs were harvested 20 h after CLP. PE-CD31$^+$ cells were isolated from lung single-cell suspensions by FACS and analyzed for ZBP1 expression by qPCR **(A)** and Western blotting (B). n=6/group. *$p < 0.05$ vs. sham. $^{\#}p<0.05$ vs. DLL4$^+$ neutrophils-CLP.

Supplemental Figure 2

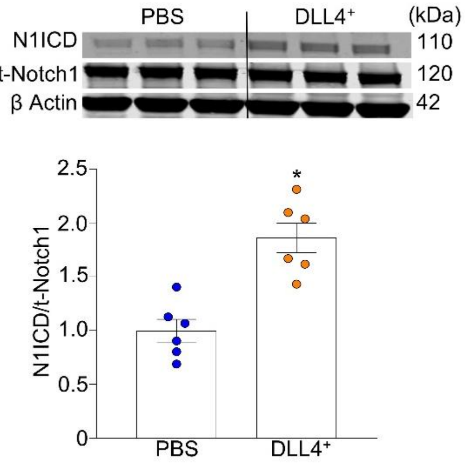

Supplemental Figure 2: DLL4+ neutrophils activate Notch1 in PVECs. PVECs (0.5×10^6) were co-cultured with FACS-sorted DLL4+ (0.5×10^6) neutrophils or PBS. After 16 h, **(A-B)** Western blot analysis was performed to measure protein levels of activated Notch1 (N1ICD), t-Notch1. Notch1 activation was quantified as N1ICD/t-Notch1. n=6/group. *$p < 0.05$ vs. PBS. t, total.

Supplemental Figure 3

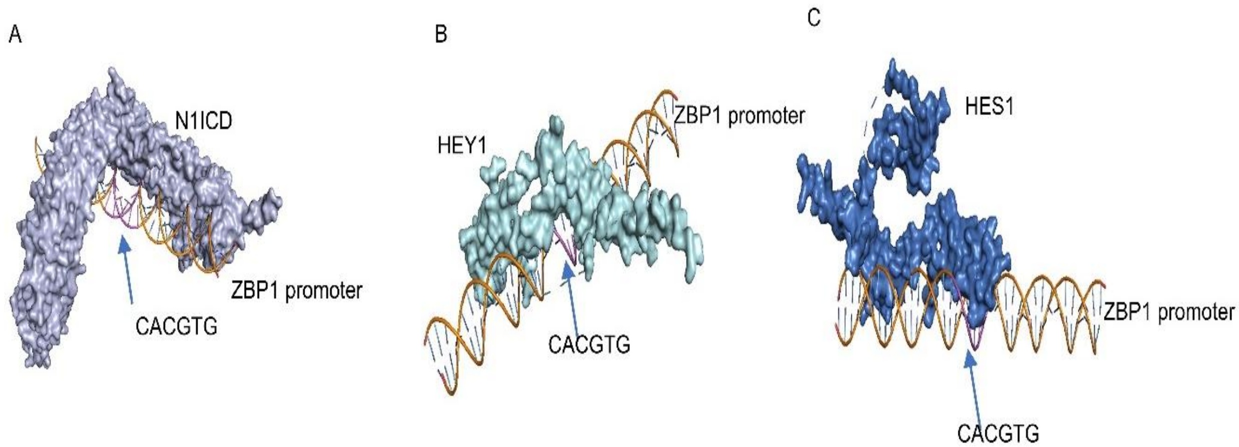

Supplemental Figure 3: Notch1 signaling activates the ZBP1 promoter. Computer model showed that Notch1 intracellular domain (N1ICD) and its target genes *HEY1* and *HES1* enhance *ZBP1* promoter activity through interaction with the CACGTG element.

Supplemental Figure 4

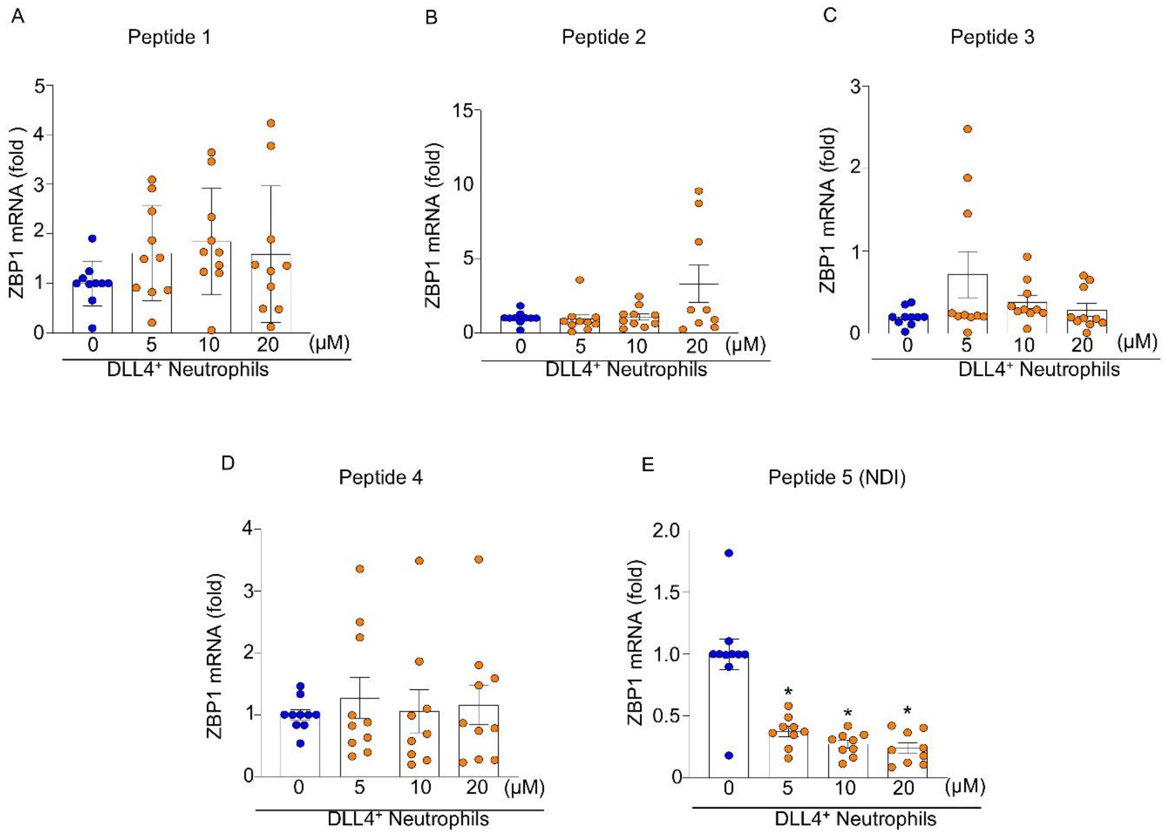

Supplemental Figure 4: 5 peptides activity detection. PVECs (0.5×10^6/mL) were co-culture with sorted DLL4$^+$ neutrophils (0.5×10^6/mL), then were treated with different dose of 5 peptides respectively. After 16 h, mRNA was extracted from PVECs, ZBP1 mRNA was detected by RT-PCR. Data was representative of three independent experiments. n=10/group. Data was expressed as means ± SE and was analyzed using one-way ANOVA. *p < 0.05 vs. PBS. PVEC: pulmonary vascular endothelial cells; DLL4: delta-like ligand 4.

Supplemental Figure 5

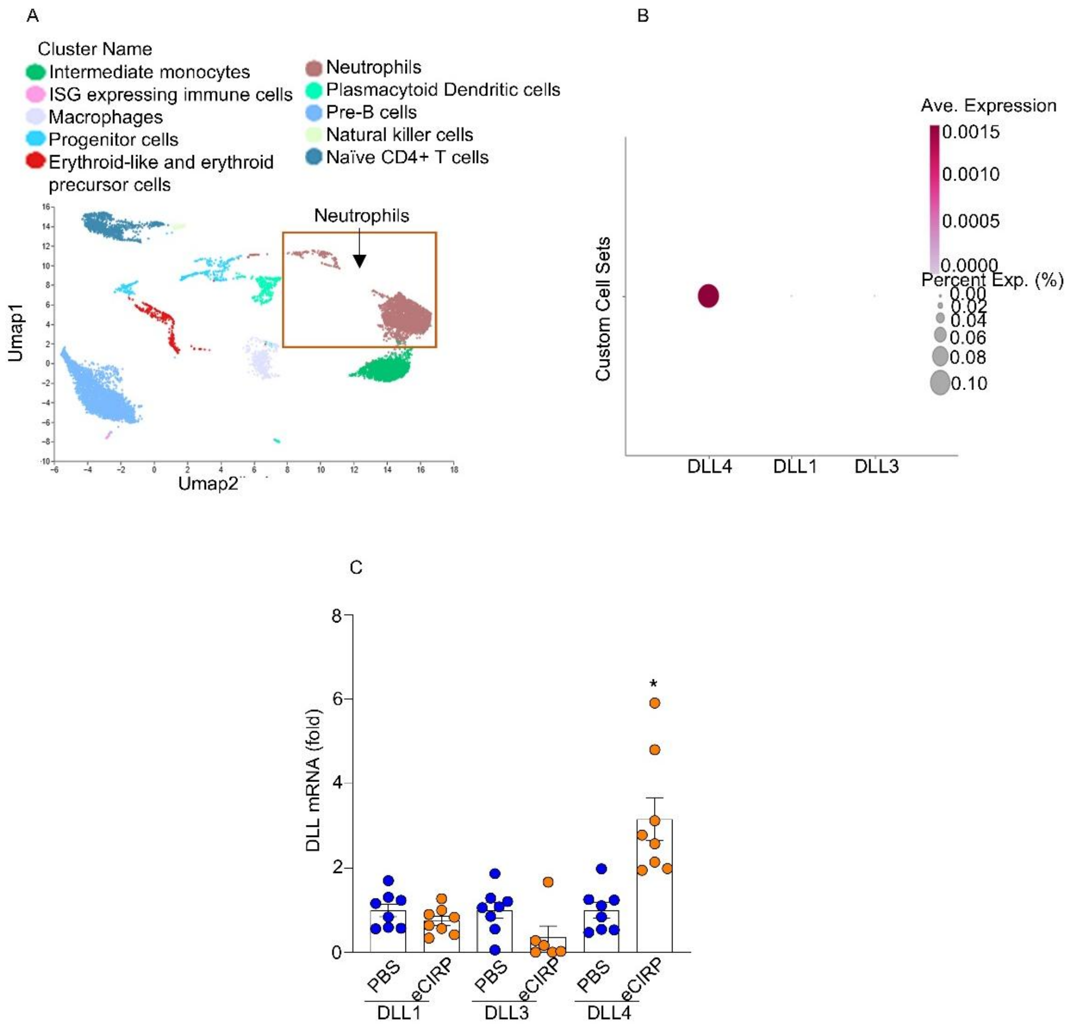

Supplemental Figure 5: Notch1 Ligand Expression in Sepsis and eCIRP-Treated Neutrophils. **(A)** scRNA-seq data on spleen and peritoneal cells of sham and septic mice were analyzed and projected with uniform manifold approximation and projection (UMAP) plots. **(B)** DLL1, DLL3, and DLL4 expression in neutrophils cluster subsets from sham control and septic mice. **(C)** BMDNs (1×10^6) were treated with eCIRP (1μg/ml) for 12h, DLL1, DLL3, and DLL4 were analyzed by qPCR. *$p < 0.05$ vs. PBS. BMDN: bone marrow drived neutrophils; eCIRP: extracellular cold inducible RNA-binding protein; DLL1, delta-like ligand 1; DLL3, delta-like ligand 3; DLL4, delta-like ligand 4.

Supplemental Table 1: Computational modeling of Notch1 target genes – ZBP1 promoter

Complex	Surface Area (Å ^{2})	Binding ($^{\Delta i}$G) Energy (Kcal/mol)	Free Energy of Dissociation (ΔG^{diss}) in Kcal/mol
mN1ICD –ZBP1 promoter	2382.0	-15.6	29.4
mHEY1 –ZBP1 promoter	2573.2	-22.8	37.0
mHES1 –ZBP1 promoter	2601.0	-20.3	34.3

Supplemental Table 2: Peptides designed for blocking Notch1-DLL4 binding

**Peptides**	**Surface Area (Å ^{2})**	**Binding ($^{\Delta i}$G) Energy (Kcal/mol)**	**Free Energy of Dissociation (ΔG^{diss}) in Kcal/mol**	**Peptides Sequences**
1	979.5	-13.0	5.7	411-DVDECALGANPCEHAGKCLN-431
2	876.7	-14.8	7.6	779-CREGFSGPNCQTNINECA-796
3	970.4	-19.7	12.0	1041-GTYKCTCPQGYTGLNCQNLV-1060
4	1123.9	-20.8	11.6	1062-WCDSAPCKNGGRCWQTNTQY-1081
5	900.8	-15.0	8.2	681-ECAGSPCHNGGTCEDGI-697

Supplemental Table 3: Computational modeling of Notch1-DLL4 peptides

Complex	Surface Area (Å ^{2})	Binding ($^{\Delta i}$G) Energy (Kcal/mol)	Free Energy of Dissociation (ΔG^{diss}) in Kcal/mol	Entropy change at dissociation ($T\Delta S^{diss}$)	N_{HB}	N_{SB}
mNotch1-DLL4-peptide 1	979.5	-13.0	5.7	9.4	4	2
mNotch1-DLL4-peptide 2	876.7	-14.8	7.6	9.1	4	1
mNotch1-DLL4-peptide 3	970.4	-19.7	12.0	9.5	4	0
mNotch1-DLL4-peptide 4	1123.9	-20.8	11.6	9.6	1	0
mNotch1-DLL4-peptide 5	900.8	-15.0	8.2	9.0	4	3

Supplemental Table 4: Mouse primers sequences

Gene	Accession No.	Forward (5'-3')	Reverse (5'-3')
TNFα	NM_013693	AGACCCTCACACTCAGATCATCTTC	TTGCTACGACGTGGGCTACA
IL-6	NM_03116	CCGGAGAGGAGACTTCACAG	CAGAATTGCCATTGCACAAC
KC	NM_03809	GCTGGGATTCACCTCAAGAA	ACAGGTGCCATCAGAGCAGT
MIP2	NM_009140	CCCTGGTTCAGAAAATCATCCA	GCTCCTCCTTTCCAGGTCAGT
ZBP1	NM_021394	ACCTTTGGCAATGTCTCCAC	TTGGATGATGATTTGGCTGA
β-actin	NM_007393	CGTGAAAAGATGACCCAGATCA	TGGTACGACCAGAGGCATACAG
DLL1	NM_007865	GCTGGAAGTAGATGAGTGTGCTC	CACAGACCTTGCCATAGAAGCC
DLL3	NM_007866	CCAGCACTGGATGCCTTTTACC	ACCTCACATCGAAGCCCGTAGA
DLL4	NM_019454	ACCTTTGGCAATGTCTCCAC	TTGGATGATGATTTGGCTGA
