# Supplementary material for: DLL4+ neutrophils promote Notch1-mediated endothelial PANoptosis to exacerbate acute lung injury in sepsis
Source: J Clin Invest. 2025 Dec 15;135(24):e194310. doi: 10.1172/JCI194310 (PMC12700550; doi:10.1172/JCI194310)

Figure 2

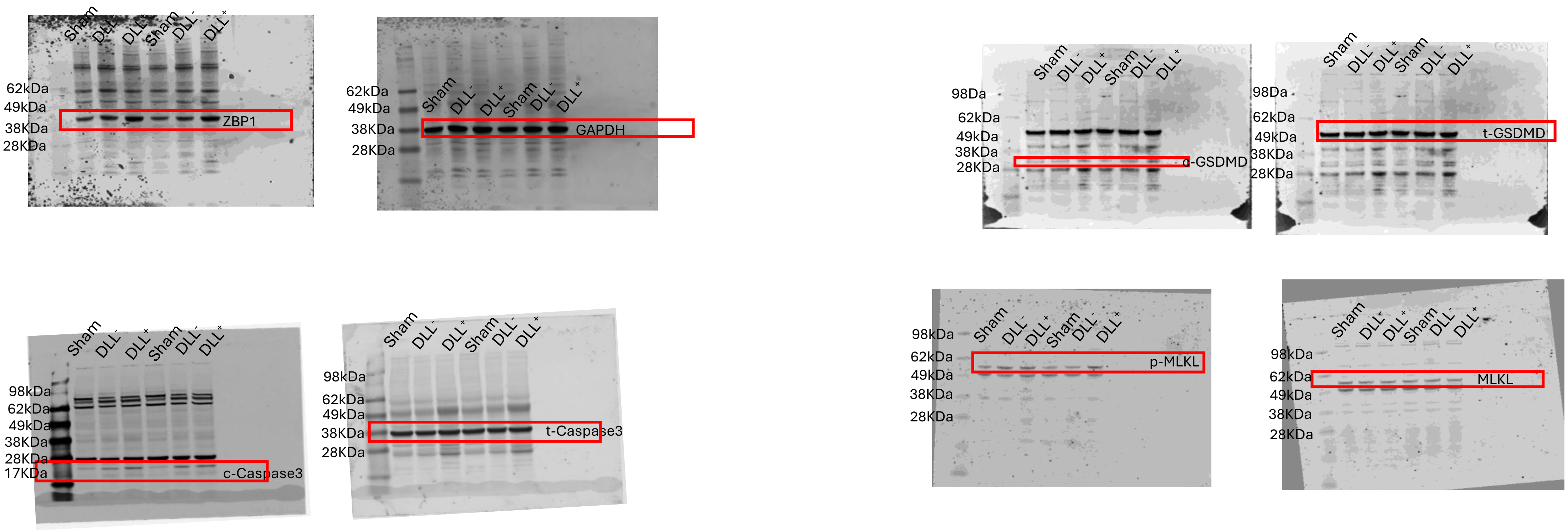
ZBP1
GAPDH
c-GSDMD
t-GSDMD
c-Caspase3
t-Caspase3
p-MLKL
MLKL
98kDa
62kDa
49kDa
38KDa
28KDa
17KDa

Figure 3

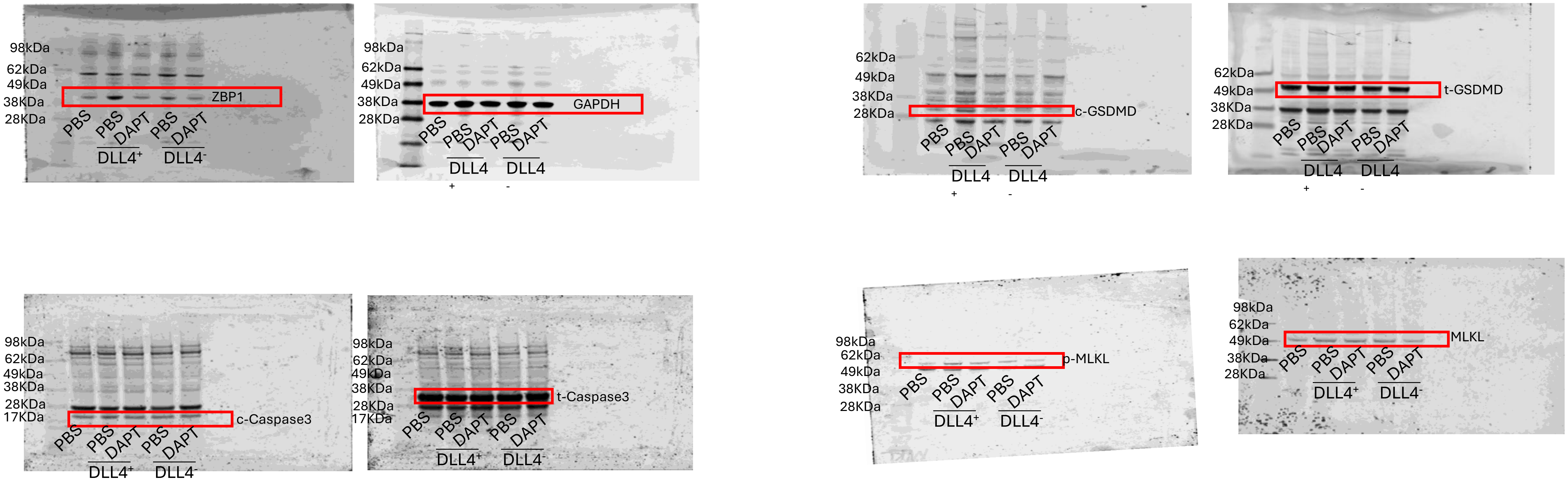
98kDa
62kDa
49kDa
38KDa
28KDa
ZBP1
PBS
PBS
DAPT
PBS
DAPT
DLL4+
DLL4-
98kDa
62kDa
49kDa
38KDa
28KDa
GAPDH
PBS
PBS
DAPT
PBS
DAPT
DLL4 +
DLL4 -
62kDa
49kDa
38KDa
28KDa
c-GSDMD
PBS
PBS
DAPT
PBS
DAPT
DLL4 +
DLL4 -
62kDa
49kDa
38KDa
28KDa
t-GSDMD
PBS
PBS
DAPT
PBS
DAPT
DLL4 +
DLL4 -
98kDa
62kDa
49kDa
38KDa
28KDa
17KDa
c-Caspase3
PBS
PBS
DAPT
PBS
DAPT
DLL4+
DLL4-
98kDa
62kDa
49kDa
38KDa
28KDa
17KDa
t-Caspase3
PBS
PBS
DAPT
PBS
DAPT
DLL4+
DLL4-
98kDa
62kDa
49kDa
38KDa
28KDa
p-MLKL
PBS
PBS
DAPT
PBS
DAPT
DLL4+
DLL4-
98kDa
62kDa
49kDa
38KDa
28KDa
MLKL
PBS
PBS
DAPT
PBS
DAPT
DLL4+
DLL4-

Figure 4

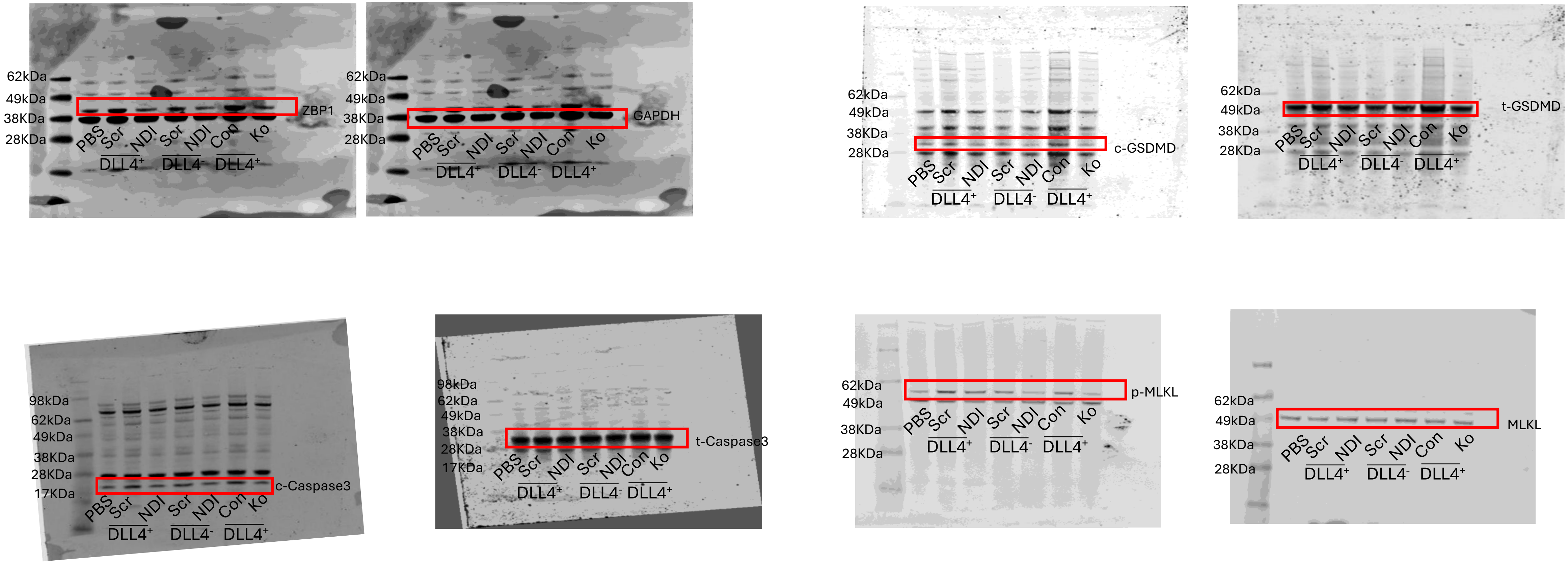
62kDa
49kDa
38KDa
28KDa
PBS Scr NDI Scr NDI Con Ko
DLL4+ DLL4- DLL4+
ZBP1
GAPDH
c-GSDMD
t-GSDMD
98kDa
17KDa
c-Caspase3
t-Caspase3
p-MLKL
MLKL

Figure 5

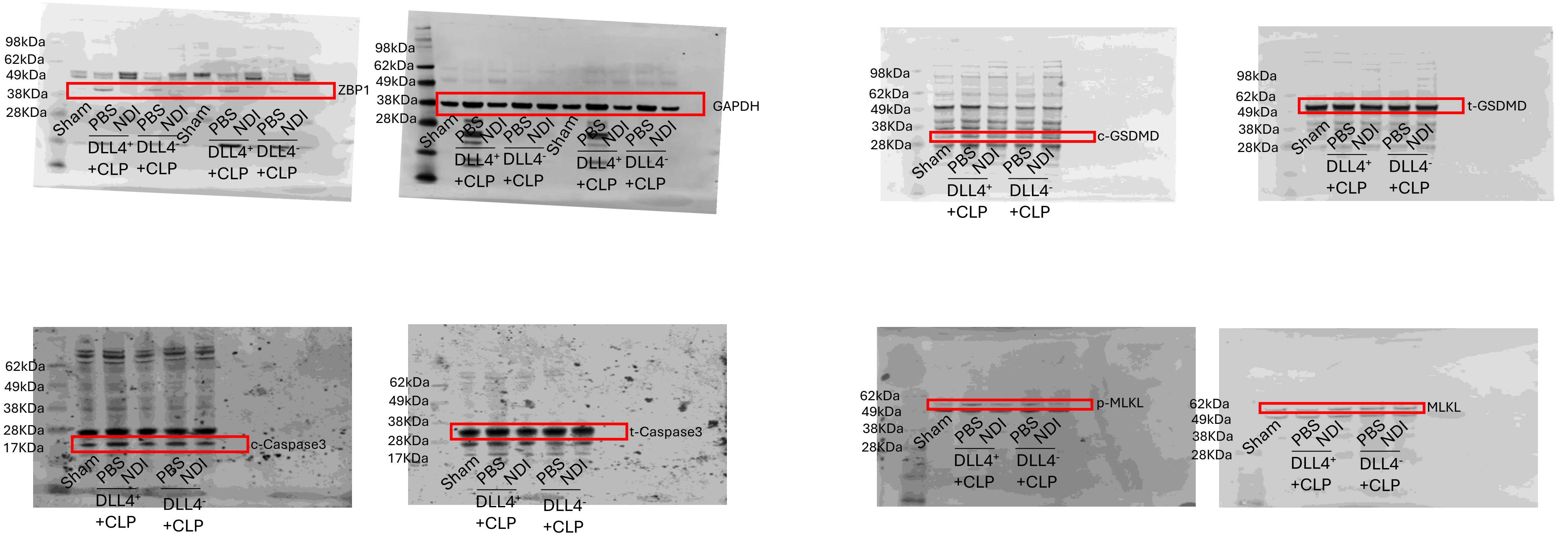

98kDa
62kDa
49kDa
38kDa
28kDa
ZBP1
Sham
PBS
NDI
DLL4+ +CLP
DLL4- +CLP
GAPDH
c-GSDMD
t-GSDMD
17KDa
c-Caspase3
t-Caspase3
p-MLKL
MLKL

Supplemental Figure 1

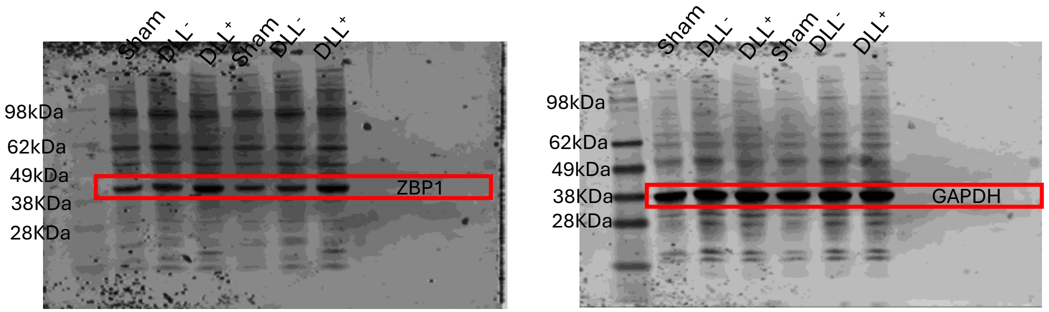

Supplemental Figure 2

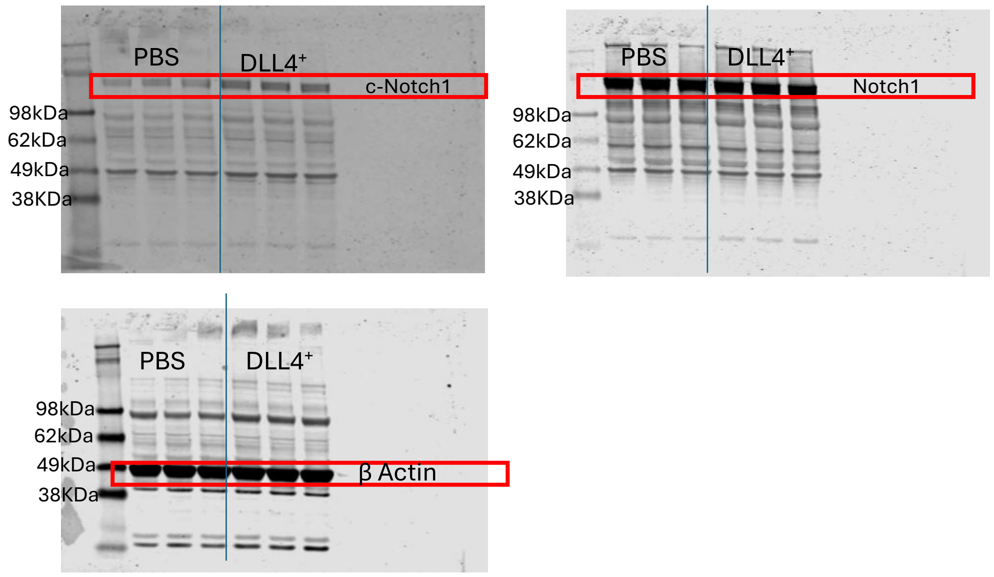

Supplement: Unedited blot and gel images [file jci-135-194310-s048.pdf]
